# Predictors of Health Care Practitioners’ Intention to Use AI-Enabled Clinical Decision Support Systems: Meta-Analysis Based on the Unified Theory of Acceptance and Use of Technology

**DOI:** 10.2196/57224

**Published:** 2024-08-05

**Authors:** Julius Dingel, Anne-Kathrin Kleine, Julia Cecil, Anna Leonie Sigl, Eva Lermer, Susanne Gaube

**Affiliations:** 1 Human-AI-Interaction Group Center for Leadership and People Management Ludwig Maximilian University of Munich Munich Germany; 2 Department of Liberal Arts and Sciences Technical University of Applied Sciences Augsburg Augsburg Germany; 3 Human Factors in Healthcare Global Business School for Health University College London London United Kingdom

**Keywords:** Unified Theory of Acceptance and Use of Technology, UTAUT, artificial intelligence–enabled clinical decision support systems, AI-CDSSs, meta-analysis, health care practitioners

## Abstract

**Background:**

Artificial intelligence–enabled clinical decision support systems (AI-CDSSs) offer potential for improving health care outcomes, but their adoption among health care practitioners remains limited.

**Objective:**

This meta-analysis identified predictors influencing health care practitioners’ intention to use AI-CDSSs based on the Unified Theory of Acceptance and Use of Technology (UTAUT). Additional predictors were examined based on existing empirical evidence.

**Methods:**

The literature search using electronic databases, forward searches, conference programs, and personal correspondence yielded 7731 results, of which 17 (0.22%) studies met the inclusion criteria. Random-effects meta-analysis, relative weight analyses, and meta-analytic moderation and mediation analyses were used to examine the relationships between relevant predictor variables and the intention to use AI-CDSSs.

**Results:**

The meta-analysis results supported the application of the UTAUT to the context of the intention to use AI-CDSSs. The results showed that performance expectancy (*r*=0.66), effort expectancy (*r*=0.55), social influence (*r*=0.66), and facilitating conditions (*r*=0.66) were positively associated with the intention to use AI-CDSSs, in line with the predictions of the UTAUT. The meta-analysis further identified positive attitude (*r*=0.63), trust (*r*=0.73), anxiety (*r*=–0.41), perceived risk (*r*=–0.21), and innovativeness (*r*=0.54) as additional relevant predictors. Trust emerged as the most influential predictor overall. The results of the moderation analyses show that the relationship between social influence and use intention becomes weaker with increasing age. In addition, the relationship between effort expectancy and use intention was stronger for diagnostic AI-CDSSs than for devices that combined diagnostic and treatment recommendations. Finally, the relationship between facilitating conditions and use intention was mediated through performance and effort expectancy.

**Conclusions:**

This meta-analysis contributes to the understanding of the predictors of intention to use AI-CDSSs based on an extended UTAUT model. More research is needed to substantiate the identified relationships and explain the observed variations in effect sizes by identifying relevant moderating factors. The research findings bear important implications for the design and implementation of training programs for health care practitioners to ease the adoption of AI-CDSSs into their practice.

## Introduction

### Background

The past decade has witnessed major advancements in the field of health care, particularly through the integration of artificial intelligence (AI). AI may be described as machines that mimic cognitive functions associated with the human mind, such as learning and problem-solving [1]. An area of progress involves the development of AI-enabled clinical decision support systems (AI-CDSSs) [2-4]. AI-CDSSs use machine learning algorithms to process vast amounts of data and provide case-specific advice to health care practitioners to aid clinical decision-making [5-7]. AI-CDSSs use clinical data both from structured (eg, laboratory test results) and unstructured (eg, clinician notes or imaging) sources. The interpretation of text-based data can be performed using natural language processing to transform text into usable data for clinical predictions [8]. In addition, deep learning models, including neural networks, can be used to generate recommendations based on image data, for example, in the detection of pneumonia from chest radiographs [9]. AI-CDSSs may improve the accuracy and efficiency of medical decision-making in several ways.

First, AI-CDSSs may offer structured rationales underpinning clinical decisions that can complement traditional care methods. This structured approach paves the way for clearer understanding, improved communication, and better tracking of the decision-making process in clinical settings [10,11]. Second, AI-CDSSs can integrate data from various sources to provide a comprehensive and personalized recommendation for every patient case [7]. Finally, AI-CDSSs promote the consistency of medical decisions. The use of AI algorithms may ensure that the same set of facts will consistently produce the same recommendations, thus minimizing harmful consequences due to human error [9].

Despite these advantages, the implementation of AI-CDSSs in clinical practice must still overcome numerous barriers. A major challenge in the deployment of AI-CDSSs is the variability in performance. This can occur when the data used to develop the AI models do not adequately represent the population for which the tool is intended. Another issue is when AI-CDSSs are not used as designed, which can be due to a range of factors, including user interface problems, lack of integration into clinical workflows, or insufficient training of health care professionals on how to use the system [7,12-14]. The resulting low performance casts doubt on the value of AI-CDSSs in assisting with clinical decision-making [12,15]. In addition, the lack of understanding of how AI recommendations are derived heightens clinicians’ reservations about using these systems [16-18]. There are also challenges related to the alignment of AI-CDSSs with existing workflows that can cause additional workload when new AI systems are incorporated into clinical procedures [7,19-21].

As the development of high-performing AI-CDSSs proceeds, understanding the factors that influence health care practitioners’ intention to use these systems becomes increasingly relevant. One of the most comprehensive theories to explain individual technology adoption is the Unified Theory of Acceptance and Use of Technology (UTAUT) [22]. The UTAUT proposes that a person’s intention to use a technology is determined by their beliefs and attitudes toward that technology, such as the perception of its performance or the perceived effort it would require to use it. The UTAUT’s comprehensive nature and its ability to account for various determinants of technology acceptance make it an appropriate model for examining the predictors of health care practitioners’ intention to use AI-CDSSs.

Research to identify predictors of the intention to use AI-CDSSs has accumulated over the past years [4,23-25]. However, the existing literature remains scattered and in need of systematic synthesis. Therefore, the overarching goal of this study was to quantitatively integrate existing studies on the predictors of health care practitioners’ intention to use AI-CDSSs. The proposed hypotheses were based on the UTAUT model and existing empirical evidence. With this meta-analysis, we make 4 major contributions to theory and practice. First, we used meta-analytic techniques to estimate the relationship between the predictors of the UTAUT and the intention to use AI-CDSSs, thus providing insights into the applicability of the UTAUT to the context of AI-CDSSs. Second, we identified additional predictors based on the existing literature and examined the relative contribution of the UTAUT and additional predictors in explaining the intention to use AI-CDSSs. With this approach, we contribute to a theoretical refinement and potential extension of the UTAUT model to the context of AI-CDSSs. Third, based on the UTAUT, we examined the role of contextual factors as moderators of the relationships between relevant predictors and use intention, thus shedding light on the conditions that influence the strength of these relationships. Finally, in line with the UTAUT model, this is the first meta-analysis that examines the role of mediators, thus allowing for a better understanding of the complex mechanisms through which use intention may be explained. The study protocol, including all hypotheses and research questions (RQs), has been preregistered through the Open Science Framework [26].

### Theory and Hypothesis Development

#### The UTAUT and the Intention to Use AI-CDSSs

The UTAUT integrates 8 former technology use theories and has become one of the most prominent technology use models [22,27]. The UTAUT has been applied to investigate factors influencing the acceptance and use of technology in different contexts, including health care [28-30]. The primary outcome measure considered in the UTAUT, alongside actual use, is the intention to use a technology [22,31,32]. Intentions are indicators of motivation and reflect the level of determination that individuals have to actually perform a certain behavior [33]. The successful deployment of any technology depends largely on the user’s intention to use it [34]. Accordingly, understanding the predictors of the intention to use AI-CDSSs may help overcome individual-level impediments thwarting the adoption of AI-CDSSs in health care.

**Figure 1:**
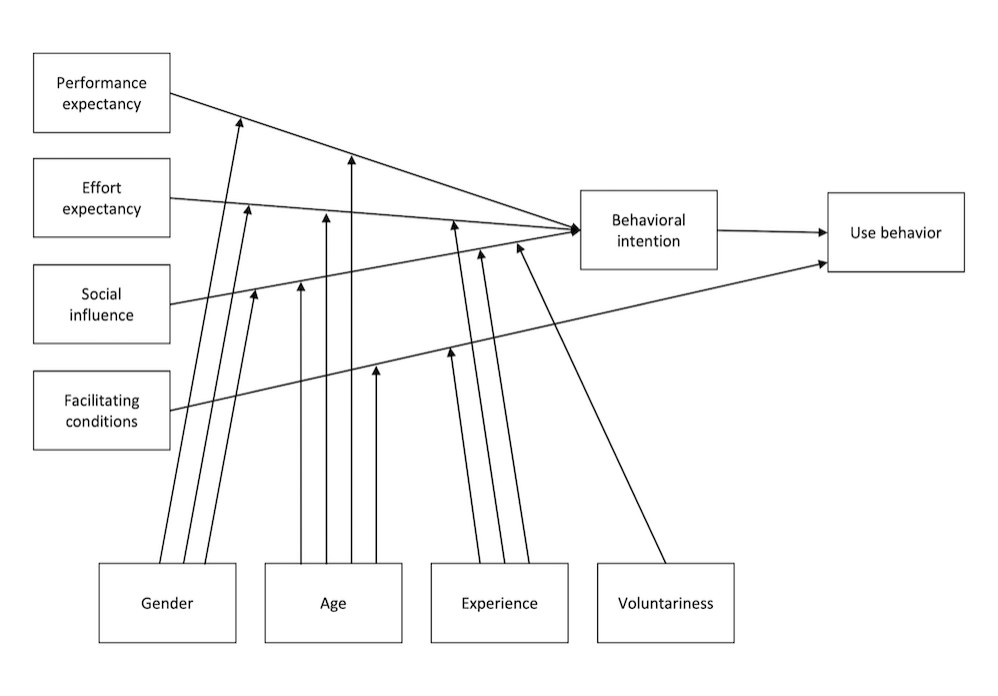
The Unified Theory of Acceptance and Use of Technology model.

The UTAUT consists of 4 core predictors of individual use intention: performance expectancy, effort expectancy, social influence, and facilitating conditions [22]. The relationships between these variables and use intention are proposed to be moderated by gender, age, experience, and voluntariness of use [22]. The UTAUT model is shown in Figure 1. All relationships included in the UTAUT were proposed as hypotheses, whereas all additional relationships and moderators that were derived based on empirical findings and other theories were proposed as RQs.

#### Predictors of the Intention to Use AI-CDSSs Based on the UTAUT

Performance expectancy refers to the extent to which individuals believe that using a technology will improve their job performance. AI-CDSSs have the potential to enhance job performance by aiding clinicians in deriving diagnoses or making treatment decisions [35]. If clinicians perceive their decisions to be improved by using AI-CDSSs, then performance expectancy will be high [36,37]. Hypothesis 1 is that performance expectancy is positively related to the intention to use AI-CDSSs.

Effort expectancy concerns the perceived ease of use of a technology. It is suggested that a system that is perceived to be easy to use is more likely to be accepted than one that is perceived to be complicated to use [22]. If, for example, the perceived effort of using an AI-CDSS in one’s existing clinical workflows is perceived to be high, health care practitioners may be less willing to use it [3,21,23]. Hypothesis 2 is that effort expectancy is positively related to the intention to use AI-CDSSs.

Social influence refers to the impact of social factors, such as the expectations and influence of peers, on an individual’s intention to use a technology. The positive relationship between social influence and the intention to use AI-CDSSs has consistently been supported in empirical studies [23,37]. For example, it has been found that medical professionals holding the belief that their colleagues, top management, and professional bodies endorse the use of AI-CDSSs in clinical settings are more willing to adopt them [37]. Hypothesis 3 is that social influence is positively related to the intention to use AI-CDSSs.

Facilitating conditions represent the organizational and technical infrastructure necessary for technology adoption [22]. It has been argued that, if users believe that the resources and support are in place to facilitate the use of AI-CDSSs, they are more likely to intend to use them [4,22,38]. Hypothesis 4.1 is that facilitating conditions are positively related to the intention to use AI-CDSSs. In addition, according to the UTAUT, there is a direct relationship between facilitating conditions and actual technology use [22]. Facilitating conditions refer to the resources and support available to use a technology, including the access to the necessary tools and knowledge. This practical aspect makes the influence of facilitating conditions on use immediate as users are more likely to use technology when they perceive a supportive environment and available resources. Unlike other predictors in the UTAUT, facilitating conditions are proposed as direct antecedents of actual use [22]. Hypothesis 4.2 is that facilitating conditions are positively related to actual use of AI-CDSSs.

#### Additional Predictors of the Intention to Use AI-CDSSs

The UTAUT has been modified, and additional predictors have been added over time to account for various settings and technologies [31,39-41]. However, a meta‐analytic review is limited to the relationships that have been studied in the literature. Following previous research and methodological best practices, we included additional predictors beyond the UTAUT in the meta-analysis that were examined in at least 3 independent samples [42,43]. Following this criterion, we identified attitude, trust, perceived risk, AI anxiety, and personal innovativeness as additional predictors of the intention to use AI-CDSSs.

Individual behavior is driven by intention, which is, in turn, a function of an individual’s attitude toward the behavior and subjective norms [31,44]. Indeed, a positive attitude toward AI-CDSSs has been identified as a relevant predictor of the intention to use AI-CDSSs [45-47]. Because the relationship between positive attitude and use intention is not included in the UTAUT, we propose the following RQ (RQ 1) to explore the relationship between positive attitude and the intention to use AI-CDSSs [22]: is there a positive relationship between a positive attitude toward AI-CDSSs and the intention to use AI-CDSSs?

Trust becomes relevant if the outcome of a situation is uncertain or the possibility of undesirable outcomes exists [48]. Trust has been argued to be a particularly relevant predictor of the intention to use AI-CDSSs due to a lack of transparency of how recommendations are derived and the high stakes of erroneous decisions in health care [23,37]. Generally, we may differentiate between initial trust as the judgment of the truster before being exposed to the trustee and knowledge-based trust that may be established after the truster has interacted with the trustee [48]. In the context of AI-CDSSs, some studies refer to initial trust in terms of beliefs in the reliability and safety of AI-CDSSs before the user has been exposed to or actively used the system [3,37,49]. An example item for initial trust is “I believe AI could provide completely accurate diagnosis assistant service” [3]. Another aspect of trust that has been explored in empirical studies is trust in different attributes of the technology, namely, its functionality (being able to do a required task), its helpfulness or benevolence (being able to provide effective help when needed or act in the best interest of the user), and its integrity (operating reliably or consistently without failing) [48,50,51]. An example item for trust in the system’s integrity regarding data security is “I trust that recommendations from the AI-powered care pathway are reliable” [51]. Because trust is not included in the UTAUT model, we propose an RQ (RQ 2) to explore whether there is a positive relationship between trust and the intention to use AI-CDSSs [22]: is there a positive relationship between trust and the intention to use AI-CDSSs?

Perceived risk is determined by the unpredictability and perceived intensity of outcomes [52]. In the context of AI-CDSSs, perceived risk refers to the perceived potential negative consequences associated with their use, including performance failure and data insecurity [4]. An example item for perceived risk of a performance failure is “There is a possibility of malfunction and performance failure, so the system might fail to deliver accurate contouring areas and could mislead my work with inaccurate contouring” [4]. Health care professionals may be reluctant to engage with new services fearing that their perceived risk may result in negative user experience or even harm to them or their patients [53]. Different forms of perceived risk have been found to be negatively associated with the intention to use AI-CDSSs [4,37,53,54]. For example, it has been found that performance and legal risk associated with AI-CDSSs are negatively related to the intention to use AI-CDSSs [37]. Because perceived risk is not included in the UTAUT model, we propose the following RQ (RQ 3) to investigate whether perceived risk is negatively associated with the intention to use AI-CDSSs [22]: is there a negative relationship between perceived risk and the intention to use AI-CDSSs?

AI anxiety encompasses general fears and insecurities regarding AI technology. It represents an intuitive, negative affective reaction to AI technologies, for example, based on the fear of making mistakes [55,56]. AI anxiety is often measured using the AI anxiety scale [22]. An example item is “I feel apprehensive about using the system.” If health care professionals experience anxiety in using AI-CDSSs, their intention to use them is presumably low. Indeed, AI anxiety has been identified as a negative predictor of the intention to use AI in health care [24]. However, because AI anxiety is not included as a predictor of use intention in the UTAUT, we propose the following RQ (RQ 4) to explore whether AI anxiety is negatively associated with the intention to use AI-CDSSs [22]: is there a negative relationship between AI anxiety and the intention to use AI-CDSSs?

Personal innovativeness describes an individual’s readiness to experiment with and embrace a new technology [57]. Those demonstrating a high degree of personal innovativeness have greater capabilities and, therefore, demonstrate greater readiness to use a new technology [58,59]. Indeed, there is empirical evidence for a positive link between personal innovativeness and the intention to use AI-CDSSs [3,36]. RQ 5 is as follows: is there a positive relationship between personal innovativeness and the intention to use AI-CDSSs?

#### The Relationship Between AI-CDSS Use Intention and Actual Use

The UTAUT proposes that an individual’s intention to use a technology is the main predictor of its actual use [22]. However, this relationship has not yet been extensively researched in the context of AI-CDSSs. The limited investigation of actual use may be attributed to the restricted number of AI-CDSSs implemented in clinical practice [60]. Nonetheless, some evidence indicates that use intention predicts the actual use of AI-CDSSs [4,47]. RQ 6 is as follows: what is the relationship between the intention to use AI-CDSSs and their actual use?

#### The Relative Contribution of the UTAUT Predictors and Additional Predictors in Explaining AI-CDSS Use Intention

Existing empirical research has explored the extent to which the UTAUT predictors account for variance in technology use intention [61]. For example, performance expectancy has often emerged as the strongest predictor of use intention [62-64]. Other research has found that trust has a stronger effect on the intention to use AI-CDSSs than performance expectancy [37]. As the roles of the UTAUT and additional predictors in explaining the intention to use AI-CDSSs remain unclear, we propose the following RQ (RQ 7): what is the relative contribution of the UTAUT predictors and additional predictors in explaining the intention to use AI-CDSSs?

#### Moderators of the Relationships Between UTAUT Predictors and the Intention to Use AI-CDSSs

The relationships between UTAUT predictors and use intention are proposed to be moderated by age, gender, user experience with the system, and voluntariness of using the system [22]. First, it has been suggested that younger workers prioritize extrinsic rewards such as improved job performance, thus exhibiting a stronger relationship between performance expectancy and technology use intention [22]. In contrast, it has been suggested that older workers generally face greater software challenges and are more likely to place increased relevance on social influences. Accordingly, they may rely more on effort expectancy and social influence when deciding to use a technology [22]. Hypothesis 5 is that the relationship between (1) performance expectancy and the intention to use AI-CDSSs becomes weaker and the relationships between (2) effort expectancy and (3) social influence and the intention to use AI-CDSSs become stronger with increasing age.

Second, the impact of performance expectancy on use intention is expected to be stronger among men, whereas the relationships between effort expectancy and social influence and use intention would be more pronounced among women [22]. Hypothesis 6 is that the relationship between (1) performance expectancy and the intention to use AI-CDSSs is stronger for men and the relationships between (2) effort expectancy and (3) social influence and the intention to use AI-CDSSs are stronger for women.

Third, according to the UTAUT, limited experience increases the strength of the relationship between effort expectancy and social influence and use intention because individuals with limited experience tend to overestimate the challenges associated with using a new technology and their opinions are more susceptible to social influence [22]. In contrast, as experience increases, facilitating conditions have been proposed to exhibit a greater impact on actual technology use as more experienced users know better in terms of how to take advantage of facilitating conditions when using the system [22]. Hypothesis 7 is that the relationships between (1) effort expectancy and (2) social influence and intention to use AI-CDSSs become weaker with increasing experience and the relationship between (3) facilitating conditions and actual use of AI-CDSSs becomes stronger with increasing experience.

Finally, the UTAUT distinguishes between voluntary (ie, individuals decide themselves whether to use a technology) and mandatory (eg, the use of a technology is mandated by the supervisor) adoption settings [22]. It has been suggested that social influence affects use intention in mandatory situations more because relevant others have the capacity to either incentivize desired actions or penalize noncompliance [22]. Hypothesis 8 is that the relationship between social influence and the intention to use AI-CDSSs is stronger in mandatory adoption settings.

In addition to the UTAUT moderators, we investigated the influence of additional contextual moderators that are studied in the literature, namely, occupation, type of AI-CDSS, and culture. All additional moderators were selected based on a comprehensive preliminary review of the literature. First, health care practitioners may work in different contexts requiring them to complete different tasks. These differences may influence their perceptions, beliefs, and attitudes toward AI-CDSSs [24,53]. For instance, one study found differences in the relationship between social influence and perceived risk and use intention between clinicians (eg, surgery and orthopedics) and nonclinicians (eg, radiologists and pathologists). Specifically, for nonclinicians, social influence positively predicted the intention to use AI-CDSSs, whereas perceived risk did not emerge as a significant predictor. In contrast, among clinicians, the reverse pattern was observed [53]. Second, the type of AI-CDSS likely influences practitioners’ use intention. Specifically, health care practitioners may place greater emphasis on the effectiveness and safety of treatment AI-CDSSs compared to diagnostic AI-CDSSs as an erroneous treatment decision is associated with more severe consequences [24]. Finally, cultural differences may influence the intention to use AI-CDSSs in health care [65,66]. For example, one study found perceived ease of use to be a more relevant predictor of the intention to use IT among Taiwanese compared to American physicians [66]. Accordingly, we propose the following RQ (RQ 8): do (1) the practitioner’s occupation, (2) the type of AI-CDSS, and (3) the cultural background moderate the relationship between UTAUT predictors and the intention to use AI-CDSSs?

Finally, we investigated the influence of methodological moderators such as publication year and the scale used to measure AI-CDSS use intention. In a meta-analysis based on the UTAUT, it was found that some effect sizes were stronger in more recent studies [61]. Moreover, while most studies use the intention to use scale introduced by Venkatesh et al [22], some studies use self-developed scales to measure use intention [25,36]. RQ 9 is as follows: do (1) publication year and (2) the use intention scale used moderate the relationship between UTAUT predictors and the intention to use AI-CDSSs?

#### Performance and Effort Expectancy as Mediators of the Relationship Between Facilitating Conditions and the Intention to Use AI-CDSSs

According to the UTAUT, the effect of facilitating conditions on use intention may be explained through performance and effort expectancy [67]. That is, if the required support infrastructure is provided, a person would perceive the system to be both high performing and easy to use, which, in turn, positively influences their intention to use it. Indeed, effort expectancy has been found to fully mediate the relationship between facilitating conditions and use intention [67]. Accordingly, we propose the following RQ (RQ 10) to investigate the mediating role of performance and effort expectancy: is the relationship between facilitating conditions and intention to use AI-CDSSs mediated through performance and effort expectancy?

#### Overview of the Hypotheses and RQs

**Figure 2:**
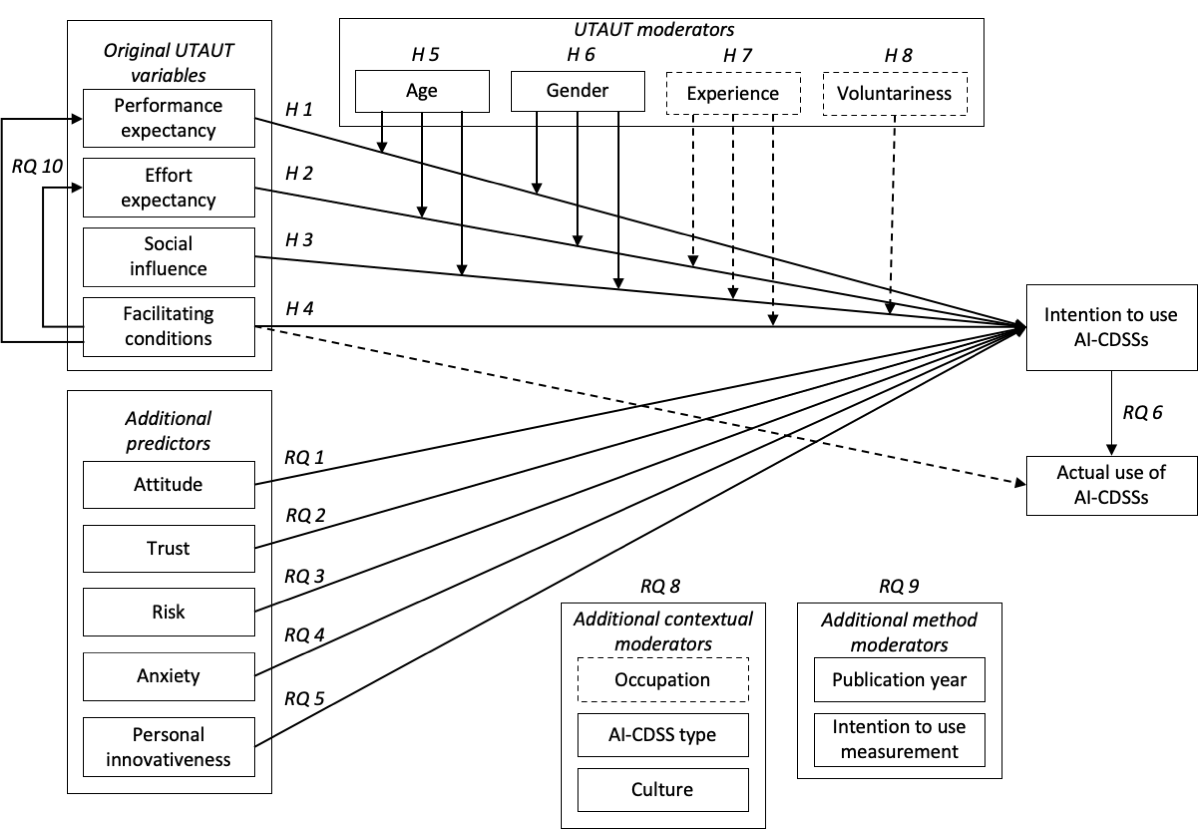
The proposed research model. The dashed lines represent preregistered hypotheses and research questions (RQs) that could not be investigated due to the limited number of available independent samples (<3). RQ 7 is omitted from the figure as it refers to the relative weight analysis. AI-CDSS: artificial intelligence–enabled clinical decision support system; H: hypothesis; UTAUT: Unified Theory of Acceptance and Use of Technology.

Figure 2 shows all hypotheses and RQs. We omitted the relationship between facilitating conditions and actual use of AI-CDSSs (hypothesis 4.2) as well as the moderators experience (hypothesis 7), voluntariness (hypothesis 8), and occupation (RQ 8.1) from the analyses (see the dashed lines in Figure 2) due to the limited number of available independent samples (<3). All deviations from the preregistration are presented in Table S1 in Multimedia Appendix 1 [3,4,22-25,34,36-38,45,47,49,53,54,56,57,68-83].

## Methods

### Inclusion and Exclusion Criteria

To qualify for inclusion, the following criteria had to be met. First, studies had to be published in English. Second, studies had to include AI-CDSSs. The second inclusion criterion was fulfilled if (1) one of the following terms—“artificial intelligence,” “AI,” “machine learning,” “deep learning,” or “deep neural networks”—was used to describe the technology [84] and (2) the technology was referred to as a clinical decision support system or it was described as providing recommendations regarding the diagnosis, treatment, or prognosis of health issues [6]. We included studies if AI-CDSSs were mentioned alongside other AI-enabled functionalities [85]. This led to the exclusion of studies that investigated the use intention of other health care technologies, such as telemedicine [86] or the Internet of Medical Things [87]. Notably, one study examined the intention to use explainable and nonexplainable AI-CDSSs in the same sample [38]. Because only one other study examined explainable AI [45], we included only the data for the nonexplainable AI-CDSSs. Third, studies had to include a measure of the intention to use AI-CDSSs as defined in the UTAUT [22], including self-developed scales based on the UTAUT scale. Fourth, studies had to be empirical. This led to the exclusion of nonempirical studies such as reviews or case studies [88]. Fifth, studies had to measure at least one predictor of the intention to use AI-CDSSs. Sixth, studies had to measure use intention among a sample of health care practitioners or medical students based on the list of health professionals by the World Health Organization [89]. Table S2 in Multimedia Appendix 1 shows a detailed overview of the inclusion criteria per included study.

### Search Strategy and Data Extraction

This meta-analysis was conducted in accordance with the PRISMA (Preferred Reporting Items for Systematic Reviews and Meta-Analyses) guidelines to ensure comprehensive and transparent reporting [90]. We used 5 steps to search for relevant data. First, relevant scientific articles, dissertations, and theses were searched using the electronic databases Embase, MEDLINE, ProQuest, PsycINFO, and Web of Science between October 15, 2022, and January 5, 2023. In total, 2 follow-up searches were conducted on May 2, 2023, and November 7, 2023. The search string was developed based on the participants, intervention, comparators, and outcome framework [91]. The framework was adapted to fit the research purpose, resulting in a 3-tiered search term including the population (health care professionals), technology (AI-CDSSs), and outcome (use intention) of interest. An overview of the search terms is presented in Table S3 in Multimedia Appendix 1. We used the search terms to search titles, abstracts, and keywords. We conducted follow-up searches in Google Scholar using the following search string: (“health care”), AND (“Artificial Intelligence”) AND (“UTAUT”). Second, we conducted forward searching of studies citing the seminal article by Venkatesh et al [22] via Google Scholar and backward searches of review articles [92-96]. Third, abstracts of relevant conference proceedings, including the *Conference on Computer-Supported Cooperative Work and Social Computing*, the *Conference on Human Factors in Computing Systems*, and the *Institute of Electrical and Electronics Engineers*, were searched. Fourth, we sent requests for unpublished articles and data using the mailing list of the German Psychology Association. Finally, authors of articles included in the meta-analysis were contacted and asked for unpublished data sets. No additional unpublished data were obtained.

We reached out to authors when critical information was needed to decide on the inclusion of a study or details essential for the meta-analytic synthesis, such as a correlation table, were missing. From the 24 authors contacted to procure missing information, we successfully obtained 6 data sets. These data sets were used to derive the missing information, for instance, to calculate missing correlations between variables of interest.

Figure S1 in Multimedia Appendix 1 shows the PRISMA diagram with the number of studies identified, included, and excluded, along with reasons for exclusion. The studies from the literature search were assessed following a 3-stage approach. First, titles were screened to identify relevant articles. Second, the abstracts of the remaining articles were reviewed. Third, full article texts were reviewed. As a result of a review of 107 full texts, 17 (15.9%) studies met the inclusion criteria (k=18 independent samples; N=3871).

Following the approach of previous meta-analyses, we only included relationships that were identified in a minimum of 3 separate samples [43,97]. We grouped overlapping variables into construct categories (see Table S4 in Multimedia Appendix 1 for definitions of superordinate constructs and subconstructs. Studies from both the primary and the follow-up literature search were coded by 2 researchers each (AK and SG for the primary search and JD and AK for the follow-up search). Any conflicts in the coding were resolved in weekly consensus meetings. In addition, in line with approaches to ensure accuracy in coding established in previous meta-analyses [98], a random sample of 56% (10/18) of the independent samples was recoded by JC and AS. We included agreement on correlations, reliabilities, and moderator categories into the assessment of interrater agreement. Overall interrater agreement was high (94.7%). Notably, no disagreements were observed regarding correlations. Some mistakes in the coding of reliabilities occurred during recoding due to referencing an incorrect line from the source document. The final code sheet used for the analyses is available on request from the Corresponding Author.

### Meta-Analytic Procedures

All analyses were conducted using RStudio (Posit Software, PBC) [99] using the R packages *psychmeta* [100] and *metaSEM* [101].

#### Bivariate Relationships

To examine the bivariate relationship between the 4 core constructs of the UTAUT (hypotheses 1-4) and the additional predictors (RQs 1-5) with the intention to use AI-CDSSs, a random-effects meta-analysis was conducted [102]. Effect sizes were based on Pearson product-moment correlations. Composites were calculated if multiple measures of the same construct were reported for the same sample [102]. Specifically, a variance-weighted composite (across measures of the same construct) was calculated for each independent sample to combine multiple measures of the same construct into a single effect size per independent sample [102]. Sampling errors were corrected using sample size–weighted correlations. Measurement errors were corrected based on the Cronbach α [102]. In addition to the sample size–weighted correlation (*r*) and sample size–weighted and reliability-corrected correlation (*r_c_*), the 95% CI and 80% credibility interval (CR) for *r_c_* were reported. Finally, we reported the correlation between observed effects and the influence of the study design artifacts.

#### Relative Weight Analysis

We conducted relative weight analyses to capture the contribution of the correlated predictors [103]. Specifically, we calculated multivariate meta-analytic regression models based on the pooled correlation matrices to explore the incremental value of the UTAUT predictors and additional predictor variables in explaining the intention to use AI-CDSSs. We used the harmonic mean of the sample size across the correlations considered as the sample size for the estimated regression models [104]. In relative weight analysis, raw relative weights are calculated to reflect the proportion of variance explained in the outcome that is attributed to each of the predictors, whereas rescaled relative weights reflect the percentage of the variance that is explained by each predictor variable [105,106].

#### Moderation Analyses

Moderator analyses were carried out for constructs that were represented in a minimum of 56% (10/18) of the independent samples to ensure adequate coverage of moderator categories [107]. A total of 5 constructs met this minimum cutoff and were considered for the moderation analyses (ie, performance expectancy, effort expectancy, social influence, trust, and perceived risk). We interpreted categorical moderator effects if each of the levels included ≥3 independent samples. Age was coded as the mean age of study participants, and gender was coded as the percentage of women in the sample. For the type of AI-CDSS, 3 categories were initially identified: diagnostic decision support systems, treatment decision support systems, and systems that combined both diagnostic and treatment decision support. However, the *treatment decision support systems* category had to be excluded from the moderator analysis because of the low number of independent samples focusing on this type of AI-CDSS (2/18, 11%). Culture was operationalized based on the individualism versus collectivism dimension of the country comparison tool by Hofstede [108,109]. A higher score denotes stronger individualism. The publication year was coded chronologically. Finally, the scale used to measure the intention to use AI-CDSSs was coded as a categorical moderator. We differentiated between studies using the scale by Venkatesh et al [22] and studies using self-developed scales. We conducted moderation analyses that were not preregistered as part of exploratory analyses.

#### Mediation Analysis

To test RQ 6, correlation-based meta-analytic structural equation modeling [110] based on the 2-stage structural equation modeling approach [111,112] was performed. In the first step, the sample size–weighted and reliability-corrected bivariate correlation matrices for each independent sample were pooled together. In 2-stage structural equation modeling, the total sample size is used for the estimation of the meta-analytic structural equation model [112]. In the second step, a path model was fitted to the pooled correlation matrix.

## Results

### Study Characteristics

The overall mean age of the participants was 36.2 (SD 13.5; range 21-53) years, and 48.7% were female. A total of 41% (7/17) of the studies focused on diagnostic AI-CDSSs, 12% (2/17) focused on treatment AI-CDSSs, 24% (4/17) focused on treatment and diagnostic AI-CDSSs, and 24% (4/17) focused on unspecific AI-CDSSs. In total, 65% (11/17) of the studies were conducted in Asia (6/11, 55% in China), 18% (3/17) were conducted in Europe, 6% (1/17) were conducted in the United States, and 12% (2/17) were conducted worldwide in English-speaking countries.

### Meta-Analytic Results

In the following sections, we report sample size–weighted and reliability‐corrected correlations (*r_c_*) for the relationships between relevant antecedent variables and AI-CDSS use intention. In line with Cohen [113], we classified our reported effects as weak (*r_c_*=0.1), moderate (*r_c_*=0.3), and strong (*r_c_*=0.5).

#### Bivariate Relationships

The results of bivariate meta-analytic analyses are shown in Table 1. The UTAUT predictors performance expectancy (*r*c=0.66, 95% CI 0.59-0.73), effort expectancy (*r*c=0.55, 95% CI 0.43-0.67), social influence (*r*c=0.66, 95% CI 0.59-0.72), and facilitating conditions (*r*c=0.66, 95% CI 0.42-0.90) exhibited a strong positive relationship with the intention to use AI-CDSSs. The findings support hypotheses 1 to 3 and 4.1. The relationship between facilitating conditions and actual use was not investigated in a sufficient number of independent samples (k<2). Accordingly, we could not address hypothesis 4.2. Regarding the additional predictors beyond the UTAUT, attitude (*r*c=0.63, 95% CI 0.52-0.73), trust (*r*c=0.73, 95% CI 0.63-0.82), and innovativeness (*r*c=0.54, 95% CI 0.43-0.64) exhibited strong positive relationships, confirming RQs 1, 2, and 5. Perceived risk (*r*c=–0.21, 95% CI –0.35 to –0.08) was weakly negatively related to use intention, supporting RQ 3. Although the estimate for AI anxiety was strong and negative (*r*c=–0.41), the 95% CI included 0 (–0.98 to 0.15). Accordingly, we cannot conclude that AI anxiety is related to use intention, thus not supporting RQ 4. The 80% CRs for effort expectancy (0.27-0.83), facilitating conditions (0.33-0.99), and AI anxiety (–0.81 to –0.01) were wide, suggesting the presence of moderators [101,112]. Finally, the intention to use AI-CDSSs was strongly positively related to the actual use of AI-CDSSs, confirming RQ 6 (3/18, 17% of independent samples; N=478; *r*=0.75; *r_c_*=0.85, SD 0.09, 95% CI 0.63-1.00, 80% CR 0.70-1.00; correlation between observed effects and the influence of the study design artifacts=0.44).

**Table 1:** Bivariate relationships between predictor variables and artificial intelligence–enabled clinical decision support system use intention (N=18).

Predictor variable	Independent samples, n (%)	Cumulative sample size, N	*r* ^a^	*r*_*c*_^b^ (SD; 95% CI)	80% CR^c^	Correlation between *r* and statistical artifacts
Performance expectancy	16 (89)	3295	0.59	0.66 (0.13; 0.59 to 0.73)	0.50 to 0.82	0.39
Effort expectancy	15 (83)	3058	0.49	0.55 (0.22; 0.43 to 0.67)	0.27 to 0.83	0.28
Social influence	15 (83)	3058	0.57	0.66 (0.12; 0.59 to 0.72)	0.52 to 0.80	0.46
Facilitating conditions	6 (33)	1048	0.57	0.66 (0.23; 0.42 to 0.90)	0.33 to 0.99	0.25
Attitude	9 (50)	2048	0.51	0.63 (0.14; 0.52 to 0.73)	0.45 to 0.80	0.43
Trust	10 (56)	1840	0.66	0.73 (0.13; 0.63 to 0.82)	0.55 to 0.90	0.35
Perceived risk	10 (56)	2428	–0.19	–0.21 (0.18; –0.35 to –0.08)	–0.45 to 0.02	0.39
Anxiety	3 (17)	391	–0.37	–0.41 (0.23; –0.98 to –0.15)	–0.81 to –0.01	0.38
Innovativeness	5 (28)	843	0.47	0.54 (0.09; 0.43 to 0.64)	0.46 to 0.61	0.81

^a^Sample size–weighted correlation.

^b^Sample size–weighted and reliability-corrected correlation.

^c^CR: credibility interval.

#### Relative Weight Analysis

It was not possible to explore all 9 predictors in a single relative weight analysis because they were not investigated together in a sufficient number of independent samples (Table S5 in Multimedia Appendix 1). Accordingly, to answer RQ 7, we analyzed 1 model with only the UTAUT predictors (Table 2) and 4 separate extension models consisting of 5 to 6 predictors (Table 3). In the initial model with only the UTAUT predictors, the combined effects of performance expectancy, effort expectancy, social influence, and facilitating conditions explained 50% of the total variance in the intention to use AI-CDSSs. Performance expectancy was the dominant predictor, accounting for 31% of the total variance explained, followed by social influence (28%), facilitating conditions (26%), and effort expectancy (15%). In the extension models, trust emerged as the most influential overall predictor of use intention (between 29% and 35% of the total variance explained). In all 3 models including trust, performance expectancy was the second most influential predictor (between 19% and 24% of the total variance explained). Facilitating conditions (between 20% and 25%) and social influence (between 14% and 21%) consistently explained additional variance in all extension models. In the extension models including trust and perceived risk as well as trust and anxiety, the regression estimate of effort expectancy became negative. Finally, AI anxiety and perceived risk negatively predicted use intention and accounted for 10% (AI anxiety) and 2% (perceived risk) of the total variance explained.

**Table 2:** Multiple regression models and relative weights for the Unified Theory of Acceptance and Use of Technology predictors^a^.

Predictor	*B*^b^ (SE)	*t* test (*df*)	*P* value	Raw RW^c^	RS^d^ RW (%)
Performance expectancy	0.31 (0.02)	13.97 (1732)	<.001	0.16	31.19
Effort expectancy	0.08 (0.02)	3.56 (1732)	<.001	0.08	15.2
Social influence	0.27 (0.02)	12.29 (1732)	<.001	0.14	27.91
Facilitating conditions	0.21 (0.02)	9.33 (1732)	<.001	0.13	25.7

^a^*F_4,1732_*=429.28 (*P*<.001); *R*^2^=0.498.

^b^Regression estimate.

^c^RW: relative weight.

^d^RS: rescaled.

**Table 3:** Multiple regression models and relative weights for the Unified Theory of Acceptance and Use of Technology (UTAUT) and additional predictors.

Predictor			*B*^a^ (SE)		*t* test (*df*)		*P* value		Raw RW^b^		RS^c^ RW (%)
**UTAUT extension (attitude and perceived risk; *F*_6,1284_=222.31; *P*<.001; *R*^2^=0.509)**											
	Performance expectancy	0.25 (0.03)		9.36 (1284)		<.001		0.12		24	
	Effort expectancy	0.05 (0.03)		2.04 (1284)		.04		0.06		12.14	
	Social influence	0.17 (0.03)		6.40 (1284)		<.001		0.10		20.54	
	Facilitating conditions	0.28 (0.03)		10.82 (1284)		<.001		0.13		25.31	
	Attitude	0.13 (0.03)		5.02 (1284)		<.001		0.08		15.91	
	Perceived risk	–0.04 (0.02)		–2.20 (1284)		.03		0.01		2.09	
**UTAUT extension (trust and innovativeness; *F*_5,1305_=308.50; *P*<.001; *R*^2^=0.542)**											
	Performance expectancy	0.22 (0.03)		8.77 (1305)		<.001		0.12		22.72	
	Effort expectancy	0.05 (0.02)		2.14 (1305)		.03		0.06		11.57	
	Social influence	0.19 (0.03)		7.62 (1305)		<.001		0.11		20.4	
	Trust	0.39 (0.03)		15.40 (1305)		<.001		0.19		35.04	
	Innovativeness	0.04 (0.02)		1.56 (1305)		.12		0.06		10.26	
**UTAUT extension (trust and perceived risk; *F*_6,1556_=389.61;*P*<.001; *R*^2^=0.600)**											
	Performance expectancy	0.18 (0.02)		8.40 (1556)		<.001		0.11		18.76	
	Effort expectancy	–0.06 (0.02)		–2.65 (1556)		.01		0.05		8.81	
	Social influence	0.09 (0.02)		3.87 (1556)		<.001		0.09		15.66	
	Facilitating conditions	0.32 (0.02)		14.99 (1556)		<.001		0.13		22.03	
	Trust	0.42 (0.02)		19.79 (1556)		<.001		0.20		33	
	Perceived risk	–0.05 (0.02)		–2.80 (1556)		.01		0.01		1.74	
**UTAUT extension (trust and anxiety; *F*_6,843_=241.15; *P*<.001; *R*^2^=0.632)**											
	Performance expectancy	0.23 (0.03)		8.15 (843)		<.001		0.12		19.25	
	Effort expectancy	–0.11 (0.03)		–3.92 (843)		<.001		0.05		7.24	
	Social influence	0.07 (0.03)		2.44 (843)		.02		0.09		14.13	
	Facilitating conditions	0.31 (0.03)		11.43 (843)		<.001		0.13		20.44	
	Trust	0.38 (0.03)		13.48 (843)		<.001		0.18		28.68	
	Anxiety	–0.20 (0.02)		–8.73 (843)		<.001		0.06		10.26	

^a^Regression estimate.

^b^RW: relative weight.

^c^RS: rescaled.

#### Moderation Analyses

Table 4 shows the results of the meta-regression for continuous moderators. Regarding age, older participants showed a weaker relationship between social influence and use intention (*B*=–0.01, 95% CI –0.01 to –0.00), thus *contradicting* hypothesis 5.3, according to which this effect would become stronger with increasing age. The moderation effect is shown in Figure S2 in Multimedia Appendix 1. Age did not moderate any other relationship, thus not confirming hypotheses 5.1 and 5.2. Gender did not moderate any of the relationships, thus not confirming hypotheses 6.1, 6.2, and 6.3. Experience and voluntariness of use were not investigated in a sufficient number of independent samples. Accordingly, we were unable to address hypotheses 7 and 8. Cultural individualism (RQ 8.3) as a contextual moderator that was measured continuously did not influence any of the relationships. Finally, publication year (RQ 9.1) as a methodological moderator that was measured continuously did not influence any of the relationships.

**Table 4:** Results of the meta-regression (N=18).

Predictor variable and moderator		Independent samples per moderator, n (%)	*B* (SE; 95% CI)	*P* value
**Performance expectancy**				
	Age	4 (22)	<0.01 (<0.01; –0.01 to 0.01)	.56
	Gender (percentage women)	16 (89)	<0.01 (<0.01; –0.00 to 0.00)	.88
	Individualism	14 (78)	<0.01 (<0.01; –0.00 to 0.00)	.66
	Publication year	16 (89)	0.02 (0.03; –0.03 to 0.07)	.42
**Effort expectancy**				
	Age	4 (22)	<0.01 (0.01; –0.02 to 0.02)	.97
	Gender (percentage women)	15 (83)	<0.01 (<0.01; –0.01 to 0.00)	.63
	Individualism	13 (72)	<0.01 (<0.01; –0.00 to 0.00)	.95
	Publication year	15 (83)	0.03 (0.04; –0.06 to 0.09)	.68
**Social influence**				
	Age	4 (22)	–0.01 (<0.01; –0.01 to –0.00)	.03
	Gender (percentage women)	15 (83)	<0.01 (<0.01; –0.00 to 0.01)	.09
	Individualism	13 (72)	<0.01 (<0.01; –0.00 to 0.00)	.56
	Publication year	15 (83)	0.02 (0.02; –0.02 to 0.07)	.21
**Trust**				
	Age	3 (17)	<0.01 (0.01; –0.02 to 0.02)	.90
	Gender (percentage women)	10 (56)	<0.01 (<0.01; –0.01 to 0.00)	.88
	Individualism	9 (50)	<0.01 (<0.01; –0.00 to 0.00)	.91
	Publication year	10 (56)	–0.04 (0.03; –0.10 to 0.03)	.24
**Perceived risk**				
	Gender (percentage women)	10 (56)	<0.01 (<0.01; –0.01 to 0.01)	.64
	Individualism	9 (50)	<0.01 (<0.01; –0.00 to 0.01)	.21
	Publication year	8 (44)	–0.02 (0.04; –0.11 to 0.06)	.60

The Wald-type pairwise comparisons for each level of categorical moderators are presented in Table 5. We could not investigate RQ 8.1 because information about occupations was not provided in a sufficient number of independent samples. Regarding RQ 8.2, the type of AI-CDSS (diagnostic AI-CDSSs versus diagnostic and treatment AI-CDSSs) did not moderate the relationship between performance expectancy and use intention nor did it moderate the relationship between social influence and use intention. However, the positive relationship between effort expectancy and use intention was stronger for diagnostic AI-CDSSs than for AI-CDSSs that combined diagnostic and treatment recommendations (mean difference=–0.31, 95% CI –0.58 to –0.04). Finally, regarding RQ 9.2, we observed no differences between studies using the scale by Venkatesh et al [22] and those using other measures.

**Table 5:** Wald-type pairwise comparisons of categorical moderators (N=18)^a^.

Predictor variable		Independent samples for moderator level 1, n (%)	Independent samples for moderator level 2, n (%)	*F* test (*df*)	*r_c1_^b^*	*r_c2_^c^*	Mean difference (95% CI)
**AI-CDSS^d^ type: diagnostic and treatment AI-CDSSs (level 1) compared to diagnostic AI-CDSSs (level 2)**							
	Performance expectancy	4 (22)	7 (39)	1.48 (*3, 3*)	0.62	0.71	–0.09 (–0.31 to 0.13)
	Effort expectancy	4 (22)	6 (33)	7.15 (*3, 3*)	0.41	0.72	–0.31 (–0.58 to –0.04)
	Social influence	4 (22)	6 (33)	6.97 (*3, 3*)	0.59	0.72	–0.14 (–0.28 to <0.01)
	Trust	3 (17)	4 (22)	0.09 (*2, 3*)	0.70	0.72	–0.02 (–0.28 to 0.24)
**Use intention scale:** **other scales (level 1) compared to the scale by Venkatesh et al [22] (level 2)**							
	Performance expectancy	10 (56)	6 (33)	0.07 (*1, 10*)	0.66	0.68	–0.02 (–0.18 to 0.15)
	Effort expectancy	9 (50)	6 (33)	0.48 (*1, 9*)	0.59	0.51	0.08 (–0.18 to 0.35)
	Social influence	9 (50)	6 (33)	0.82 (*1, 9*)	0.64	0.70	–0.06 (–0.21 to 0.09)
	Trust	5 (28)	5 (28)	0.01 (*1, 6*)	0.73	0.72	0.01 (–0.19 to 0.20)
	Perceived risk	7 (39)	3 (17)	2.41 (*1, 2*)	–0.16	–0.37	0.21 (–0.10 to 0.53)

^a^Moderator analysis for constructs assessed in at least 10 independent samples.

^b^Sample size–weighted and reliability-corrected correlation for moderator level 1.

^c^Sample size–weighted and reliability-corrected correlation for moderator level 2.

^d^AI-CDSS: artificial intelligence–enabled clinical decision support system.

#### The Mediating Role of Performance and Effort Expectancy in the Relationship Between Facilitating Conditions and AI-CDSS Use Intention

The role of performance and effort expectancy as mediators of the relationship between facilitating conditions and intention to use AI-CDSSs (RQs 10.1 and 10.2) was analyzed by fitting 2 separate mediation models. The results are shown in Table 6. Performance expectancy and effort expectancy mediated the relationship between facilitating conditions and the intention to use AI-CDSSs (indirect effect for performance expectancy: *B*=0.20, 95% CI 0.12-0.34; indirect effect for effort expectancy: *B*=0.21, 95% CI 0.09-0.37).

**Table 6:** Mediation models with performance and effort expectancy as mediators.

Path				*B*^a^ (95% CI)
**Mediator: performance expectancy**				
	**Direct effects**			
		Facilitating conditions→performance expectancy	0.38 (NA^b^ to 0.54)	
		Performance expectancy→use intention	0.53 (0.36 to 0.70)	
		Facilitating conditions→use intention	0.29 (–0.01 to 0.57)	
	**Indirect effect**			
		Facilitating conditions→performance expectancy→use intention	0.20 (0.12 to 0.34)	
**Mediator: effort expectancy**				
	**Direct effects**			
		Facilitating conditions→effort expectancy	0.48 (0.35 to 0.62)	
		Effort expectancy→use intention	0.43 (0.17 to 0.68)	
		Facilitating conditions→use intention	0.29 (–0.04 to 0.61)	
	**Indirect effect**			
		Facilitating conditions→effort expectancy→use intention	0.21 (0.09 to 0.37)	

^a^Regression estimate.

^b^NA: the lower bound of the CI could not be estimated.

### Sensitivity Analysis

To assess the robustness of the meta-analytic findings, we used cumulative meta-analysis. This approach involves conducting a sequence of iterative meta-analyses, with each analysis adding an effect size for a specific relationship. Effect sizes are added in order of decreasing precision, meaning that the initial effect sizes added represent the most accurate population effect size estimates. If less precise studies tend to skew the meta-analytic estimates, this will be observable as a shift in cumulative results when these studies are included [106]. The results of the cumulative meta-analyses are shown in Figure S3 in Multimedia Appendix 1. A total of 5 “drifts” were identified, and all relationships drifted toward stronger effects as less precise studies were added, indicating an overestimation of the true effect. However, meaningful differences were not observed for any of the relationships after half the studies were added compared to after all the studies were added (Table S6 in Multimedia Appendix 1). Accordingly, we conclude that none of the drifts influenced the meta-analytic conclusions.

## Discussion

### Summary of Findings and Implications for Future Research

The primary goal of the meta-analysis was to gain a better understanding of the predictors of intention to use AI-CDSSs among health care practitioners based on the UTAUT and its extensions. The results of the meta-analysis provide empirical support for the applicability of the UTAUT to the context of AI-CDSSs. As predicted, performance expectancy, effort expectancy, social influence, and facilitating conditions were positively related to the intention to use AI-CDSSs. These findings are largely in line with the findings of UTAUT meta-analyses in other fields [27,32,40,61]. We provide a summary of the main findings for our hypotheses and RQs in Tables 7 and 8.

The results of relative weight analyses showed that all 4 UTAUT predictors together explained 50% of the variance in use intention among health care practitioners, reaffirming the relevance of the UTAUT predictors in the context of AI-CDSSs. Among the UTAUT predictors, performance expectancy emerged as the most relevant, accounting for 31% of the total explained variance, followed by social influence (28%), facilitating conditions (26%), and effort expectancy (15%). In most UTAUT research, performance expectancy is more relevant than effort expectancy, possibly because performance expectancy is inherently connected to the primary motives behind technology use [32,40]. That is, it directly relates to the perceived benefits that users expect to gain from using a technology [32,40]. Effort expectancy refers to the expected ease of using a technology [22]. While important, the ease of use may become a secondary consideration if the technology does not meet the primary performance-related objectives. In other words, users might be willing to overcome a steeper learning curve if they believe the payoff in performance is worthwhile [63]. This could explain why performance expectancy accounts for a higher percentage of the variance in technology acceptance and use intentions compared to effort expectancy. Overall, the findings of this meta-analysis reflect a common finding in technology acceptance research where the anticipated improvement in performance is often found to be a stronger driver of user acceptance than the anticipated effort to learn and use the technology [32,40,61,63].

**Table 7:** Results of the investigation of the hypotheses (N=18).

Hypothesis	Independent samples, n (%)	Effect size (95% CI)	Result	Main findings
1	16	0.66^a^ (0.59 to 0.73)	Supported	Performance expectancy is positively related to the intention to use AI-CDSSs^b^.
2	15	0.55^a^ (0.43 to 0.67)	Supported	Effort expectancy is positively related to the intention to use AI-CDSSs.
3	15	0.66^a^ (0.59 to 0.72)	Supported	Social influence is positively related to the intention to use AI-CDSSs.
4.1	6	0.66 (0.42 to 0.90)	Supported	Facilitating conditions are positively related to the intention to use AI-CDSSs.
4.2	<3	—^c^	—	Not enough independent samples (<3) to examine the relationship between facilitating conditions and actual use of AI-CDSSs
5.1	4	<0.01^d^ (–0.01 to 0.01)	Not supported	The relationship between performance expectancy and intention to use AI-CDSSs does not become weaker with increasing age.
5.2	4	<0.01^d^ (–0.02 to <0.01)	Not supported	The relationship between effort expectancy and intention to use AI-CDSSs does not become stronger with increasing age.
5.3	4	−0.01^d^ (–0.01 to <–0.01)	Not supported	The relationship between social influence and intention to use AI-CDSSs becomes weaker with increasing age.
6.1	16	<0.01^d^ (<–0.01 to <0.01)	Not supported	The relationship between performance expectancy and intention to use AI-CDSSs is not stronger for men.
6.2	15	<0.01^d^ (–0.01 to <0.01)	Not supported	The relationship between effort expectancy and intention to use AI-CDSSs is not stronger for women.
6.3	15	<0.01^d^ (<–0.01 to 0.01)	Not supported	The relationship between social influence and intention to use AI-CDSSs is not stronger for women.
7.1-7.3	<3	—	—	Not enough independent samples (<3) to examine experience as a moderator
8	<3	—	—	Not enough independent samples (<3) to examine mandatory versus voluntary adoption setting as a moderator

^a^Sample size–weighted and reliability-corrected correlation.

^b^AI-CDSS: artificial intelligence–enabled clinical decision support system.

^c^Not applicable.

^d^Regression estimate.

Among the UTAUT predictors, effort expectancy and facilitating conditions had the widest CRs (0.56 and 0.66, respectively), suggesting the presence of moderating influences [102,114]. For example, previous research suggests that radiologists, accustomed to complex machines and heavy workloads, may be willing to invest effort into learning how to use new technology if it reduces their workload, indicating a moderating influence of occupation on the relationship between effort expectancy and use intention [4,37]. In addition, the strength of the relationship between effort expectancy and use intention has been shown to differ between AI-CDSSs for feedback versus decision support [24].

In addition to the core UTAUT variables, we identified attitude, trust, perceived risk, AI anxiety, and personal innovativeness as predictors of the intention to use AI-CDSSs. Although all the included studies (17/17, 100%) reported a negative relationship between AI anxiety and use intention, the CI for AI anxiety included 0. This lack of an observed relationship may be due to the low sample sizes (the total sample size was 391) and the resulting high uncertainty in the true effect. Interestingly, in the relative weight analyses, trust proved to be a more relevant factor than performance expectancy in explaining variance in the intention to use AI-CDSSs. The relevance of trust may be explained by the lack of transparency in how AI recommendations are generated coupled with the high stakes associated with clinical decision-making [115]. Indeed, research has suggested that even highly efficient AI-CDSSs may face resistance in clinical applications if health care practitioners do not trust the system’s safety [116-118]. The findings of this meta-analysis align with those of research advocating for the inclusion of trust in the UTAUT model [116].

**Table 8:** Results of the investigation of the research questions (N=18).

Research question	Independent samples, n (%)	Effect size (95% CI)	Result	Main findings
1	9	0.63^a^ (0.52 to 0.73)	Answered	Positive attitude toward AI-CDSSs^b^ is positively related to intention to use AI-CDSSs.
2	10	0.73^a^ (0.63 to 0.82)	Answered	Trust is positively related to intention to use AI-CDSSs.
3	10	–0.21^a^ (–0.35 to –0.08)	Answered	Perceived risk is negatively related to intention to use AI-CDSSs.
4	3	–0.41^a^ (–0.98 to –0.15)	Answered	AI^c^ anxiety is negatively related to intention to use AI-CDSSs.
5	5	0.54^a^ (0.43 to 0.64)	Answered	Personal innovativeness is positively related to intention to use AI-CDSSs.
6	3	0.85^a^ (0.63 to 1.00)	Answered	The intention to use AI-CDSSs is positively related to their actual use.
7	—^d^	See Table 3	Partially answered	See Table 3
8.1	<3	—	—	Not enough independent samples (<3) to examine occupation as a moderator
8.2	4 for moderator level 1; 6 for moderator level 2	0.31^e^ (–0.58 to –0.04)	Answered	The positive relationship between effort expectancy and use intention was weaker for diagnostic and treatment AI-CDSSs (moderator level 1) than for diagnostic AI-CDSSs (moderator level 2).
8.3	13-14	See Table 4	Answered	Cultural background (individualism) does not moderate the relationship between performance expectancy (14 independent samples), effort expectancy (13 independent samples), and social influence (13 independent samples) and intention to use AI-CDSSs.
9.1	15-16	See Table 4	Answered	Publication year does not moderate the relationship between performance expectancy (16 independent samples), effort expectancy (15 independent samples), and social influence (15 independent samples) and intention to use AI-CDSSs.
9.2	6-10	See Table 5	Answered	There are no differences in the relationships between performance expectancy, effort expectancy, and social influence and intention to use AI-CDSSs between samples that used the scale by Venkatesh et al [22] to measure use intention (moderator level 2) and those that used other scales (moderator level 1).
10.1	16	0.20^f^ (0.12 to 0.34)	Answered	Performance expectancy mediates the relationship between facilitating conditions and intention to use AI-CDSSs.
10.2	16	0.21^f^ (0.09 to 0.37)	Answered	Effort expectancy mediates the relationship between facilitating conditions and intention to use AI-CDSSs.

^a^Sample size–weighted and reliability-corrected correlation.

^b^AI-CDSS: artificial intelligence–enabled clinical decision support system.

^c^AI: artificial intelligence.

^d^Not applicable.

^e^Mean difference between sample size–weighted and reliability-corrected correlation for moderator levels 1 and 2.

^f^Regression estimate (indirect effect).

Furthermore, this meta-analysis emphasizes the need to consider both drivers and inhibitors of the intention to use AI-CDSSs for a more comprehensive understanding of the adoption process [119]. The relative weight analyses demonstrate that AI anxiety explained approximately 10% of the variance in the intention to use AI-CDSSs after trust (29%), facilitating conditions (20%), performance expectancy (19%), and social influence (14%) and before effort expectancy (7%). The relevance of perceived risk as a predictor of use intention was small (approximately 2% after all other predictors). Risk perception is a cognitive assessment of the potential losses and gains from using AI-CDSSs, which is based on logical evaluation and can be mitigated by providing relevant information [120]. In contrast, AI anxiety is an emotional response that encompasses fears and insecurities about AI technology [121]. Accordingly, AI anxiety is less rational and more difficult to alleviate because it can be deeply rooted in concerns about AI’s impact on job security, professional autonomy, and the quality of patient care [24,122,123].

Even for relationships assessed in a substantial number of independent samples, such as performance expectancy, effort expectancy, attitude, trust, and perceived risk, the CRs were wide (>0.34), suggesting the presence of moderators [102,114]. This observation is supported by the modest amount of variance accounted for by statistical artifacts, indicating that there may be other reasons for substantial variance between individual studies [102,114]. While we considered multiple moderators suggested by the UTAUT and additional contextual and methodological moderators, we only found 2 moderation effects.

First, age moderated the relationship between social influence and use intention, with older health care practitioners exhibiting a weaker relationship between social influence and use intention. This finding does not align with the UTAUT proposing that older individuals place more relevance on the opinion of relevant others when intending to use a new technology [22]. An explanation for this discrepancy may be that practitioners value their professional independence increasingly more with age, thus relying less on the opinion of others regarding technology use as they get older. While differences in professional values and behaviors have been shown to exist between younger and older health care practitioners [124], a systematic examination of the effect of age on the relationship between social influence and the intention to use AI-CDSSs is lacking. It has to be noted that the observed moderation effect was based on only 22% (4/18) of independent samples, underscoring the need to systematically study the influence of age on the relationship between social influence and use intention.

Second, the relationship between effort expectancy and use intention was stronger for diagnostic AI-CDSSs than for devices that combined diagnostic and treatment recommendations. When clinicians assess a tool solely for diagnostic purposes, they may find it easier to anticipate the required effort to use it as the task is less complex and the outcome is more direct. This clear understanding may strengthen the relationship between effort expectancy and use intention. Indeed, the perceived risk associated with smart devices has been found to negatively influence the relationship between effort expectancy and use intention [125]. The multifaceted nature of combined tools may make it more challenging for clinicians to evaluate the effort needed to understand and use them. This uncertainty possibly leads to a weaker relationship between effort expectancy and use intention as clinicians may not be able to adequately assess the effort required, thus not being able to use it as a source of information when it comes to indicating their intention to use it. Future research is needed to investigate the moderating influence of device type.

The results of the mediation analyses indicate that the relationship between facilitating conditions and use intention may be explained through effort expectancy and performance expectancy. This finding aligns with the UTAUT proposing that, when performance and effort expectancy are considered, facilitating conditions lose their importance in predicting use intention [22]. An explanation for the relevance of effort expectancy as a mediator may be that issues related to the support infrastructure, a critical aspect of facilitating conditions, are also conceptually addressed by effort expectancy [22]. That is, if health care organizations establish the appropriate support infrastructure, the effort required to use AI-CDSSs becomes lower [22,54]. Similarly, if a user perceives that the technology is supported by adequate facilitating conditions, they may be more likely to believe in the performance benefits when using the system, explaining the mediating role of performance expectancy.

### Practical Implications

Performance expectancy and trust emerged as the 2 most relevant predictors of AI-CDSS use intention, suggesting that measures targeted toward health care practitioners’ beliefs in the performance and trustworthiness of AI-CDSSs may be effective in enhancing their intention to use them. However, the consistently positive link between performance expectancy and use intention also suggests that health care institutions need to take measures to deter the perception of low-performing systems as high performing, which could potentially cause more harm than benefit [126]. Health care practitioners require transparent communication regarding the performance and limitations of AI-CDSSs alongside adequate training to ensure their correct use. In addition, regulatory bodies such as the Food and Drug Administration need to ensure that available AI-CDSSs meet certain safety and performance standards [84,127,128]. Adequate policies and oversight in these contexts may ensure a balance between the adoption and safe application of AI-CDSSs in health care decision-making.

Trust in technology is a multifaceted construct including users’ perceptions of a system’s functionality, helpfulness or benevolence, and integrity [48,49]. Consequently, actions taken to enhance performance expectancy may not be sufficient for building trust in a system [116]. If organizations aim to improve health care practitioners’ trust in AI-CDSSs, they need to address the various facets relevant to trust in technology. This includes dealing with ethical issues related to data privacy and the potential misuse of AI-CDSSs as well as addressing the lack of transparency and explainability in AI-generated recommendations [129,130]. For example, trust has been associated with the system’s capability to explain its decision-making process, emphasizing the role of explainable AI as a path to building trust in AI-CDSSs [116,131]. In addition, regulatory strategies should be designed to promote and maintain trust in AI-CDSSs along with safe patient outcomes. This might include the use of postmarket surveillance systems to monitor the performance of deployed AI-CDSSs over time, which has been suggested as a method for identifying and mitigating issues of utility and safety in real-world clinical settings [132,133]. Developers can integrate user-centered design principles to tailor AI-CDSSs to the needs and workflows of specific clinical specialties and roles. The early inclusion of user feedback may facilitate the development of user-friendly AI-CDSSs and increase trust in these systems [134,135]. Health care administrators may foster practitioners’ trust by providing training programs to increase familiarity with the technology and by designing evaluation metrics that can monitor system performance and user satisfaction [136].

Social influence has been demonstrated to be a relevant predictor of health care practitioners’ intentions to use AI-CDSSs, particularly among younger professionals. Institutions aiming to adopt AI-CDSSs can leverage the important role of social influence by establishing a culture that values technological advancements and by engaging key opinion leaders to advocate and exemplify the use of these systems. In addition, trainings can be structured not only to educate but also to establish a shared understanding and a community of practice that positively reinforces the application of AI-CDSSs [137,138]. By addressing the social aspects of technology acceptance, health care institutions can ensure that their investment in AI is met with a user base that is both competent and willing to integrate these tools into their daily practice.

The importance of facilitating conditions underscores the need for health care organizations to provide a supportive infrastructure that simplifies the integration of AI-CDSSs into existing workflows. For instance, the provision of training programs, allowing health care practitioners to gain firsthand experience, and setting up accessible support teams ready to address system-related issues can considerably boost health care practitioners’ intention to use such systems [23,139].

AI anxiety emerged as a barrier to the intention to use AI-CDSSs in the relative weight analysis. Therefore, hospitals and other health care institutions should consider measures to counteract any irrational negative emotional reactions to AI before and during the integration of AI-CDSSs into clinical workflows. A potential method to mitigate AI anxiety involves increasing medical staff involvement in the development process [123] or providing more training opportunities to increase their exposure to AI-enabled devices, thus reducing irrational fears [137].

### Limitations and Implications for Future Research

This meta-analysis is not without limitations. First, this study offers insights into the predictors of use intention as the key determining factor of actual use. However, some health care practitioners may express intention to use AI-CDSSs but be hesitant when it comes to their actual implementation. Few studies included in the meta-analysis (3/17, 18%) examined the predictors of actual use, underscoring the need for additional research on predictors of the actual use of AI-CDSSs [4,48].

Second, we were unable to explain the considerable variation in some of the effects based on moderator analyses. We could not evaluate 3 UTAUT moderators—experience with AI-CDSSs, voluntariness of use, and occupation—owing to insufficient samples incorporating these variables. In addition, although all studies including AI anxiety (3/17, 18%) reported negative relationships with use intention, the CI of the meta-analytic estimate included 0 due to the low sample size and the associated high uncertainty in the estimate. More studies on the relationship between AI anxiety and intention to use AI-CDSSs are needed. The large CRs and the low correlations between estimates and statistical artifacts suggest the existence of moderating factors not included in the meta-analysis [102,114]. Future research should explore moderating effects such as differences in the observed relationships among health care practitioners working in different fields or roles to better understand the boundary conditions that affect the relationships between predictors and the intention to use AI-CDSSs.

Third, the 9 relevant predictors could not be examined in a single relative weight analysis. The use of multiple models with subsets of predictors is a pragmatic approach to addressing data sparsity. However, the selected approach hinders definitive conclusions regarding the importance of all considered predictors. Furthermore, innovativeness could not be assessed in the relative weight analysis due to a lack of available samples assessing this predictor. The compromises that had to be made in the relative weight analyses highlight the need for an updated meta-analysis that includes complete predictor sets.

Fourth, the insights derived from the meta-analysis are primarily confined to unspecific AI-CDSSs. Given that AI-CDSS adoption is still limited, only a handful of studies have delved into exploring predictors of the use of specific AI-CDSSs with distinctive features [4,24,38]. The results of these studies show that the attitude toward AI-CDSSs may vary depending on use cases and system features. Future research should examine the adoption of individual systems and variations in effects across different types of AI-CDSSs.

Fifth, the existing body of research on AI-CDSS adoption primarily relies on cross-sectional observational studies, with questionnaires as the main method of data collection. These studies inherently limit the establishment of causal relationships, thus underscoring the need for future research to include longitudinal or experimental designs for more robust evidence of causality. Longitudinal studies may also be used to shed light on the development of use intention and the relevance of relevant predictors over time. For example, it is possible that initial trust plays a crucial role during the implementation phase but becomes less relevant once a system has been successfully implemented.

Sixth, we selected the UTAUT as a general theoretical framework to examine the predictors of intention to use AI-CDSSs. However, there has been some criticism of the UTAUT [64,140]. For example, the UTAUT may not answer questions related to the determinants and processes involved in *value-adding* technology use [64,141,142]. We found support for the prediction that beliefs about the performance and ease of use of AI-CDSSs lead to a higher intention to use these systems. However, based on the UTAUT, whether these beliefs are well founded (ie, whether positive expectations actually lead to beneficial use because the system is indeed high performing and easily implementable) may not be resolved. Another criticism pertains to the UTAUT’s narrow viewpoint on individual use. Other models such as the nonadoption, abandonment, scale-up, spread, and sustainability framework [143] adopt a system perspective. This approach enables the examination of predictors at micro (individual technology users), meso (organizational processes and systems), and macro (national policy and wider context) levels, thereby more accurately representing the complex processes involved in technology adoption [143,144]. In addition, the UTAUT focuses on an individual’s intention to use a technology and does not fundamentally consider how well the technology fits the task it is being used for. Theories such as the task-technology fit model examine the interconnectedness between task and technological characteristics. The model delves into how features of both the assigned task and the technology at hand shape the task-technology fit, thereby influencing the overall performance and use intentions [145].

Finally, some of the predictors included in this meta-analysis, particularly the additional predictors beyond the UTAUT, may not be adequately represented using standard measurement instruments. For instance, AI anxiety has a multitude of dimensions, such as privacy violation anxiety, bias behavior anxiety, job replacement anxiety, learning anxiety, or ethics violation anxiety [146]. This meta-analysis did not distinguish between these different aspects of AI anxiety as separate predictors of AI-CDSS use intention. Similarly, trust in AI is a multifaceted construct that includes perceptions of the system’s benevolence, competence, and integrity [48,49]. Moreover, trust may refer to different aspects of an AI-CDSS, such as trust in the reliability of its predictions when being applied to different contexts, trust in legal protection if harm to patients occurs from using the AI-CDSS, and trust in data privacy [4,47,147]. More research is needed that explores the relevance of different elements of trust (ie, benevolence, competence, and integrity) and elements of the AI-CDSSs that may be trusted to different degrees (eg, reliability, legal and liability issues, and privacy concerns) for the intention to use AI-CDSSs in clinical decision-making [147].

### Conclusions

This meta-analysis underscores the relevance of the UTAUT to examine the predictors of intention to use AI-CDSSs in health care. The results indicate that performance expectancy, effort expectancy, social influence, and facilitating conditions are positively related to the intention to use AI-CDSSs among health care practitioners. The analyses further revealed the relevance of the additional predictors attitude, trust, personal innovativeness, AI anxiety, and perceived risk. The results of mediation analyses show that effort expectancy and performance expectancy explain the relationship between facilitating conditions and use intention. Despite identifying age and AI-CDSS type as moderating influences, there is scope for future research to investigate other possible moderators to explain the variability in the observed effects. While the UTAUT model provides a theoretical framework for studying health care practitioners’ intention to use AI-CDSSs, it remains relatively silent on the predictors of value-adding use of AI-CDSSs. Future research could investigate the conditions that encourage value-adding use by applying comprehensive frameworks that consider both individual and broader organizational processes (eg, clinic systems and administrative hurdles). Finally, the findings of this meta-analysis provide starting points for the development and integration of AI-CDSSs that are likely to be adopted by health care practitioners as end users.

## Appendix

Multimedia Appendix 1 Supplementary materials including tables regarding preregistration deviation , inclusion criteria per included study, search terms per database, construct and subconstruct definitions, pooled meta-analytic correlations and number of samples per correlation, results of the cumulative meta-analyses and figures regarding the PRISMA (Preferred Reporting Items for Systematic Reviews and Meta-Analyses) flowchart, age as a moderator of the social influence–use intention relationship, and results of the cumulative meta-analyses.

## Abbreviations

AI: artificial intelligence
AI-CDSS: artificial intelligence–enabled clinical decision support system
CR: credibility interval
PRISMA: Preferred Reporting Items for Systematic Reviews and Meta-Analyses
RQ: research question
UTAUT: Unified Theory of Acceptance and Use of Technology
