# Supplementary material for: Predictors of Health Care Practitioners’ Intention to Use AI-Enabled Clinical Decision Support Systems: Meta-Analysis Based on the Unified Theory of Acceptance and Use of Technology
Source: J Med Internet Res. 2024 Aug 5;26:e57224. doi: 10.2196/57224 (PMC11333871; doi:10.2196/57224)
Supplement: Multimedia Appendix 1 [file jmir_v26i1e57224_app1.pdf]

Predictors of Healthcare Practitioners' Intention to Use AI-Enabled Clinical Decision Support Systems (AI-CDSSs): A Meta-Analysis Based on the Unified Theory of Acceptance and Use of Technology (UTAUT)

Multimedia Appendix

This is a Multimedia Appendix to a full manuscript published in the J Med Internet Res. For full copyright and citation information see http://dx.doi.org/10.2196/jmir.57224

Table of Contents

Table S1. Preregistration deviations table 3
Table S2. Inclusion criteria per included study 6
Table S3. Search terms per database 10
Table S4. Construct and subconstruct definitions 11
Table S5. Pooled meta-analytic correlations and number of samples per correlation 13
Table S6. Cumulative meta-analyses 14
Figure S1. PRISMA flow chart of the study selection process 16
Figure S2. Age as a moderator of the social influence–use intention relationship 17
Figure S3. Results of cumulative meta-analyses 18

Table S1. Preregistration deviations table

<table>
<tr><th colspan="6">Deviations</th></tr>
<tr><th>#</th><th colspan="2">Details</th><th>Original Wording</th><th>Deviation Description</th><th>Reader Impact</th></tr>
<tr><td rowspan="3">1</td><td>Type</td><td>Research Q(s)</td><td rowspan="3">Research Question 4: What additional predictors beyond the UTAUT have been empirically tested and how do they contribute to explaining behavioral intention?</td><td rowspan="3">Research Questions 1 to 5 outline the additional predictors that extend beyond the UTAUT model.

Research Question 1: Is there a positive relationship between a positive attitude towards AI-CDSSs and the intention to use AI-CDSSs?

Research Question 2: Is there a positive relationship between trust and the intention to use AI-CDSSs?
Research Question 3: Is there a negative relationship between perceived risk and AI-CDSSs use intention?
Research Question 4: Is there a negative relationship between AI anxiety and the intention to use AI-CDSSs?
Research Question 5: Is there a positive relationship between personal innovativeness and the intention to use AI-CDSSs?</td><td rowspan="3">The deviation should not affect the interpretation of the meta-analytic results.</td></tr>
<tr><td>Reason</td><td>New knowledge</td></tr>
<tr><td>Timing</td><td>During data collection</td></tr>
<tr><td rowspan="3">2</td><td>Type</td><td>Research Q(s)</td><td rowspan="3">Research Question 3: What is the relative contribution of the different UTAUT predictors in explaining behavioral intention?

Research Question 5: What is the relative contribution of the</td><td rowspan="3">We combined Research Questions 3 and 5.

Research Question 7: What is the relative contribution of the UTAUT predictors and additional predictors in explaining the intention to use AI-CDSSs?</td><td rowspan="3">The deviation should not affect the interpretation of the meta-analytic results.</td></tr>
<tr><td>Reason</td><td>Better readability</td></tr>
<tr><td>Timing</td><td>Before data collection</td></tr>
</table>

			additional predictors in explaining behavioral intention?		
3	Type	Research Q(s)	Research Question 2: Do contextual (occupation, country, AI instrument) and methodological factors (behavioral intention scale) moderate the relationship between UTAUT predictors and behavioral intention?	*We rephrased the original research question by splitting it into two research questions. We added publication year as an additional moderator (addressed in "Unregistered Steps").*<br><br>Research Question 8: Do (a) the practitioner's occupation, (b) the type of AI-CDSS, and (c) cultural background moderate the relationship between UTAUT predictors and the intention to use AI-CDSSs?<br><br>Research Question 9: Do (a) publication year and (b) the use intention scale employed moderate the relationship between UTAUT predictors and the intention to use AI-CDSSs?	*The deviation should not affect the interpretation of the meta-analytic results.*
	Reason	Better readability			
	Timing	Before data collection			

Unregistered Steps

#	Details		Original Wording	Unregistered Step Description	Reader Impact
1	Type	Research Q(s)	*The new Research Question 6 was not addressed in the preregistration.*	Research Question 6: What is the relationship between the intention to use AI-CDSSs and their actual use?	*The unregistered step may affect the interpretation of the relevance of AI-CDSS use intention for actual use. We do not expect this step to influence the interpretation of other results.*
	Timing	During data collection			

2	Type	Research Q(s)	*The new Research Question 9(a) was not addressed in the preregistration.*	*Research Question 9*: Do (a) publication year and (b) the use intention scale employed moderate the relationship between UTAUT predictors and the intention to use AI-CDSSs?	*The unregistered step may affect the interpretation of the relevance of publication year as a moderator. We do not expect this step to influence the interpretation of other results.*
	Timing	During data collection			
3	Type	Research Q(s)	*The new Research Question 10(a) was not addressed in the preregistration.*	*Research Question 10*: Is the relationship between facilitating conditions and the intention to use AI-CDSSs mediated through (a) performance and (b) effort expectancy?	*The unregistered step may affect the interpretation of the relevance of performance expectancy as a mediator between facilitating conditions and AI-CDSS use intention. We do not expect this step to influence the interpretation of other results.*
	Timing	During data collection			

Table S2. Inclusion criteria per included study

Study	Inclusion criteria						
	**#1 English**	**#2a) AI or related term used to describe technology**	**#2b) technology provides decision support regarding diagnosis, treatment or prognosis of health issues**	**#2c) AI-CDSS mentioned alongside other AI-enabled health technologies**	**#3 Intention to use measurement**	**#4) Sample of healthcare professionals**	**#5) Predictor and outcome variable measured**
Wang et al. (2021)	Yes	'AI', 'deep learning'	Regarding intelligent registration, diagnosis, and treatment as well as intelligent pathological diagnosis and medical image recognition	Yes, alongside telemedicine, intelligent drug research and development, medical robots, smart wearable devices	Author's own definition	Yes, diverse sample of healthcare practitioners	Attitude, Intention to use
Tamori et al. (2022)	Yes	'AI'	Regarding diagnosis and treatment without requiring a doctor	No	Author's own definition	Yes, sample included both non-healthcare and healthcare professionals; only data from the latter was used	Performance expectancy, Effort expectancy, Social influence, Risk, Intention to use
Prakash & Das (2021)	Yes	'AI'	Regarding one or more component steps of the diagnostic process	No	Venkatesh et al. (2003)	Yes, radiologists	Performance expectancy, Effort expectancy, Social influence, Trust, Risk, Intention to use
Zhai et al. (2021)	Yes	'AI'	Regarding primary gross tumor volume and	No	Venkatesh et al. (2003)	Yes, radiologists	Performance expectancy, Effort expectancy, Social influence, Facilitating

			normal tissue contouring process				conditions, Risk, Intention to use, Acutal use
Fan et al. (2020)	Yes	'AI'	Regarding medical diagnosis	No	Author's own definition	Yes, diverse sample of healthcare practitioners	Performance expectancy, Effort expectancy, Social influence, Trust, Innovativeness, Intention to use
Pan et al. (2019)	Yes	'AI'	Regarding data analysis and decision support services (via emerging technologies such as AI and cloud computing)	Yes, alongside Internet of Things (IoT), e.g., sensors, and electronic health records	Venkatesh et al. (2003) and Miao et al. (2017)	Yes, two separate samples of healthcare practitioners (clinicians and non-clinicians)	Performance expectancy, Effort expectancy, Social influence, Attitude, Risk, Intention to use
Tran et al. (2021)	Yes	'AI'	Regarding diagnosis	No	Author's own definition	Yes, medical students	Performance expectancy, Effort expectancy, Social influence, Innovativeness, Trust, Intention to use
Cornelissen et al. (2022)	Yes	'AI'	Regarding the management of chronic diseases via care pathways that offer the right care at the right time and are continuously risk-adjusted using AI	No	Venkatesh et al. (2003)	Yes, but also includes non-healthcare practitioners (e.g., management, consultants)	Performance expectancy, Effort expectancy, Social influence, Facilitating conditions, Innovativeness, Anxiety, Trust, Intention to use
Cheng et al. (2022)	Yes	'AI'	Regarding diagnosis and treatment	No	Venkatesh et al. (2003)	Yes, diverse sample of dental healthcare practitioners	Performance expectancy, Effort expectancy, Social influence, Trust, Intention to use
Panigutti et al. (2022)	Yes	'Recurrent neural network'	Regarding patients’ future diagnoses based on their past clinical histories.	No	Spil & Schuring (2006); Venkatesh	Yes, diverse sample of healthcare practitioners	Performance expectancy, Effort expectancy, Social influence, Facilitating conditions, Attitude, Intention to use

					& Bala (2008)		
Ritter (2019)	Yes	'AI', 'deep learning'	Genomics AI, medical imaging analysis, and prognostic/ predictive analytics all support diagnosis, treatment or prognosis	Yes, alongside genomics AI, medical imaging AI, monitoring AI, prognostic/predictive analytics, research AI, robotic assistance, robotic surgery, training AI	Alshehri (2013)	Yes, physicians	Performance expectancy, Effort expectancy, Social influence, Facilitating Conditions, Trust, Risk, Anxiety, Attitude, Intention to use, Actual use
Calisto et al. (2022)	Yes	'AI'	Regarding diagnosis (during the medical imaging workflow)	No	Sohn & Kwon (2020); Ye et al. (2019)	Yes, diverse sample of healthcare practitioners"	Performance expectancy, Effort expectancy, Social influence, Facilitating conditions, Trust, Risk, Intention to use
Kleine et al. (2023)	Yes	'AI'	Regarding treatment via voice recordings and mood scores	Yes, alongside a second tool (AI-enabled psychotherapy feedback tool), but data was separate and excluded	Venkatesh et al. (2003)	Yes, psychology students	Performance expectancy, Effort expectancy, Social influence, Facilitating conditions, Trust, Anxiety, Attitude, Intention to use
Yang et al. (2023)	Yes	'AI'	Regarding diagnostic assistance and treatment recommendations	Yes, alongside knowledge query and quality control of medical orders	Rahman et al. (2017); Sohn & Kwon (2020); Wu et al. (2011)	Yes, physicians	Effort expectancy, Performance expectancy, Social influence, Innovativeness, Trust, Intention to use
Hsieh (2023)	Yes	'AI'	Regarding AI-assisted diagnosis and its application to brain magnetic resonance imaging (MRI) segmentation	No	Davis (1989)	Yes, physicians	Performance expectancy, Innovativeness, Trust, Intention to use

Eiskjaer et al. (2023)	Yes	'AI'	Regarding shared decision-making on the choice of treatment of ordinary spinal disorders	No	Venkatesh et al. (2003)	Yes, spine surgeons	Performance expectancy, Effort expectancy, Social influence, Trust, Intention to use, Actual use
Dalvi-Esfahani et al. (2023)	Yes	'Explainable AI (XAI)'	Regarding a broad spectrum of clinical decision-making, encompassing diagnosis, therapy, and pharmacotherapy management	No	Liu (2022)	Yes, diverse set of healthcare practitioners	Attitude, Risk, Intention to use

Table S3. Search terms per database

Database	Search fields	Search term
Embase, Medline, ProQuest, PsycInfo, and Web of Science	Title, abstract and keyword	('health professional*' OR ‘doctor*’ OR 'care provider*' OR 'physician*' OR 'practitioner*' OR 'clinician*' OR 'nurse*' OR 'dentist*' OR 'psychotherap*' OR 'psychiatrist*' OR 'radiologist*' OR 'medical student*' OR 'psychology student*') AND ('artificial intelligence' OR 'machine learning') AND ('behavioral use' OR 'behavioral intent* OR 'intention to use' OR 'usage' OR 'frequency of use' OR 'feature* used' OR 'time* used' OR 'duration of use' OR 'use duration' OR ‘implement*’ OR ‘adopt*’)
ACM Digital Library	Title, abstract, full text, and keywords	[All: ‘healthcare’] AND [All: ‘artificial intelligence’] AND [All: ‘use’].
Conference on Computer Supported Cooperative Work and Social Computing, Conference on Human Factors in Computing Systems. Institute of Electrical and Electronics Engineers	Abstract	[All: ‘healthcare’] AND [All: ‘artificial intelligence’] AND [All: ‘use’].
Google Scholar (Follow-up searches)	NA	[‘healthcare’], AND [‘Artificial Intelligence’] AND [‘UTAUT’]

Table S4. Construct and subconstruct definitions

Construct (bold) or subconstruct	Definition
**Proactive career behavior**	The perception that using an AI-CDSS will help attain gains in job performance (Venkatesh et al., 2003)
Career exploration	Beliefs that AI-CDSS will be useful (Tamori et al., 2022)
Perceived efficiency	The perception that AI-CDSS will help to provide services efficiently (Tamori et al., 2022)
Medical performance expectancy	The perception that using AI-CDSS will help attain gains in terms of the provided quality of care (Cornelissen et al., 2022)
Nonmedical performance expectancy	The perception that using AI-CDSS will help to attain gains in productivity, efficiency, and communication (Cornelissen et al., 2022)
Functional Value	The rational and economic evaluation of the quality of the AI-CDSS (Sheth et al., 1991)
**Effort expectancy**	The perceived ease associated with the use of the AI-CDSS (Venkatesh et al., 2003)
Perceived ease of use	The perception that using the AI-CDSS would be free of effort (Davis, 1989)
Ease of mastery	The perception that doctors could quickly master the use of the AI-CDSS in medicine (Tamori et al., 2022)
User-friendliness	The perception that doctors could easily operate the AI-CDSS in medical settings (Tamori et al., 2022)
**Social influence**	The perception that important others believe that the AI-CDSS should be used (Venkatesh et al., 2003)
Expectations of others	Perceived optimism of people around the healthcare practitioner regarding the potential of the AI-CDSS (Tamori et al., 2022)
Expectations among patients	Perceived optimism of patients regarding the potential of the AI-CDSS (Tamori et al., 2022)
**Facilitating conditions**	The perception that an organizational and technical infrastructure exists to support the use of the AI-CDSS (Venkatesh et al., 2003)
**Positive attitude**	An individual's overall positive affective reaction to using the AI-CDSS (Venkatesh et al., 2003)
Optimism	The perceived extent to which the AI-CDSS provides more control, flexibility, and efficiency (Hsieh, 2023)

**Trust**	The belief that the AI-CDSS will act cooperatively to fulfill expectations without exploiting vulnerabilities (Venkatesh et al., 2011)
Initial trust	Trust in the performance and efficacy of an unfamiliar AI-CDSS that has not been used before (McKnight, 2005)
Trust in system ability, integrity, benevolence	The belief that the AI-CDSS has the ability, integrity, and benevolence needed in providing services (Cornelissen et al., 2022)
Trust in competence, benevolence, willingness, reciprocity	Trust in system competence, benevolence, willingness, reciprocity (Gulati et al., 2018)
**Risk**	Perceived potential negative consequences associated with the use of the AI-CDSS, including performance failure, data insecurity, additional workload (Zhai et al., 2021)
Privacy concerns	The concern about the potential disclosure or sharing of personal information by the AI-CDSS with third parties without explicit consent or authorization (Brady et al., 2021)
Medico-legal risk	The concern surrounding potential legal liability arising from the use of the AI-CDSS, including inadequate protection, ambiguity in assigning responsibility for damages, and manufacturers' attempts to shield themselves from legal liability (Prakash & Das, 2021)
Performance risk	The concern regarding the performance of the AI-CDSS, including doubts about its reliability, level of benefits, potential diagnostic errors, and perceived technical immaturity (Prakash & Das, 2021)
Concern about data leakage	The level of concern regarding the potential leakage of personal data resulting from the use of the AI-CDSS (Tamori et al., 2022)
Concern about accountability and liability	The level of concern regarding who would be accountable or liable in case of accidents or errors resulting from the use of the AI-CDSS (Tamori et al., 2022)
Perceived unregulated standard	The belief that regulatory standards and guidelines to assess the AI-CDSS algorithmic safety are yet to be formalized (Esmaeilzadeh, 2020)
**AI Anxiety**	The fear and intimidation experienced by an individual during their interaction with an AI-CDSS, including fear of losing information and making irreversible mistakes (Venkatesh et al., 2003)
**Innovativeness**	The willingness of an individual to try out a new innovation (Agarwal & Prasad, 1998)

Note. The superordinate constructs displayed in bold font were used as constructs in the meta-analysis. The subconstructs were matched to the superordinate constructs.

Table S5. Pooled meta-analytic correlations and number of samples per correlation

	PE	EE	FC	SI	ATT	TR	RI	ANX	IN	BI
PE		15	6	15	6	10	9	3	5	16
EE	0.51		6	15	5	10	8	3	4	15
FC	0.46	0.55		6	3	4	4	3	1	6
SI	0.56	0.48	0.56		5	10	8	3	4	15
ATT	0.59	0.46	0.39	0.55		2	5	2	-	9
TR	0.56	0.53	0.36	0.55	*NA*		4	3	4	10
RI	-0.20	-0.23	-0.14	-0.13	-0.15	-0.15		2	-	10
ANX	-0.10	-0.36	-0.20	-0.21	*NA*	-0.30	*NA*		1	3
IN	0.48	0.60	*NA*	0.54	*NA*	0.49	*NA*	*NA*		5
BI	0.59	0.49	0.57	0.57	0.52	0.66	-0.19	-0.37	0.47	

Note. Sample size-weighted and reliability-corrected correlation (r_c) in lower triangle and number of samples per bivariate relationship (k) in upper triangle

Table S6. Cumulative meta-analyses

Study added	*k*	*N*	*r*	r_c	SD_c	95% CI	80% CR
**Effort expectancy**							
2	1	399	.31	.34	NA	.24, .43	-
6	2	744	.31	.37	0.07	-.22, .96	.27, .47
15	3	1,087	.30	.34	0.07	.16, .51	.26, .42
20	4	1,409	.40	.45	0.24	.07, .84	.07, .84
4	5	1,716	.46	.52	0.25	.21, .83	.14, .90
8	6	1,927	.47	.53	0.23	.28, .77	.19, .87
21	7	2,133	.45	.50	0.24	.28, .72	.17, .83
5	8	2,324	.46	.52	0.23	.32, .71	.19, .84
3	9	2,507	.47	.52	0.23	.35, .70	.22, .83
7	10	2,646	.46	.52	0.22	.36, .68	.23, .82
22	11	2,783	.48	.54	0.23	.39, .69	.24, .84
17	12	2,901	.48	.54	0.22	.40, .68	.25, .83
14	13	2,968	.48	.54	0.22	.41, .67	.26, .82
24	14	3,03	.49	.54	0.22	.42, .67	.26, .83
16	15	3,058	.49	.55	0.22	.43, .67	.27, .83
**Social influence**							
2	1	399	.51	.55	NA	.48, .63	-
6	2	744	.46	.55	0.01	.47, .63	.55, .55
15	3	1,087	.47	.55	0.01	.53, .57	.55, .55
20	4	1,409	.48	.56	0.02	.52, .60	.56, .56
4	5	1,716	.53	.62	0.12	.47, .76	.45, .79
8	6	1,927	.55	.53	0.12	.51, .75	.47, .79
21	7	2,133	.57	.65	0.13	.53, .77	.48, .83
5	8	2,324	.58	.66	0.12	.55, .76	.49, .82
3	9	2,507	.58	.66	0.12	.57, .75	.51, .82
7	10	2,646	.57	.66	0.12	.57, .74	.51, .81
22	11	2,783	.58	.66	0.12	.58, .74	.51, .82
17	12	2,901	.58	.66	0.12	.58, .73	.51, .81
14	13	2,968	.57	.66	0.12	.58, .73	.51, .80
24	14	3,030	.57	.66	0.12	.59, .72	.51, .80
16	15	3,058	.57	.66	0.12	.59, .72	.52, .80
**Attitude**							
1	1	404	.33	.43	NA	.32, .55	-

2	2	803	.43	.53	0.12	-.51, 1.57	.21, .85
6	3	1,148	.44	.56	0.11	.28, .83	.38, .74
23	4	1,385	.46	.58	0.11	.40, .77	.42, .75
21	5	1,591	.51	.63	0.16	.44, .83	.41, .86
25	6	1,763	.51	.63	0.14	.48, .78	.43, .83
7	7	1,902	.51	.64	0.14	.51, .77	.45, .82
17	8	2,02	.51	.62	0.14	.51, .74	.45, .80
16	9	2,048	.51	.63	0.14	.52, .73	.45, .80
**Trust**							
15	1	343	.60	.65	NA	.58, .72	-
20	2	665	.59	.64	0.01	.55, .74	.64, .64
8	3	876	.57	.62	0.06	.47, .76	.54, .69
21	4	1,082	.60	.65	0.09	.50, .79	.52, .78
5	5	1,273	.63	.69	0.14	.52, .86	.49, .89
3	6	1,456	.65	.71	0.14	.56, .85	.51, .90
22	7	1,593	.66	.72	0.14	.59, .84	.53, .90
17	8	1,711	.67	.73	0.14	.62, .84	.55, .91
14	9	1,778	.66	.73	0.13	.63, .83	.55, .90
24	10	1,840	.66	.73	0.13	.63, .82	.55, .90
**Risk**							
2	1	399	-.13	-.15	NA	-.26, -.04	-
6	2	744	-.10	-.13	0.04	-.50, .25	-.13, -.13
20	3	1,066	-.11	-.13	0.03	-.21, -.05	-.13, -.13
4	4	1,373	-.17	-.20	0.14	-.42, .03	-.41, .01
23	5	1,610	-.16	-.19	0.13	-.35, -.03	-.36, -.02
21	6	1,816	-.15	-.18	0.12	-.31, -.05	-.33, -.03
3	7	1,999	-.19	-.22	0.18	-.39, -.05	-.46, .03
25	8	2,171	-.20	-.23	0.18	-.38, -.08	-.47, .01
7	9	2,310	-.20	-.23	0.17	-.37, -.10	-.46, -.01
17	10	2,428	-.19	-.21	0.18	-.35, -.08	-.45, .02

Note. k = number of independent samples; N = cumulative sample size; r = sample size-weighted correlation; r_c = sample size-weighted and reliability-corrected correlation; SD_c = standard deviation of r_c; CI = confidence interval for rc; CR = credibility interval.

Figure S1. PRISMA flow chart of the study selection process

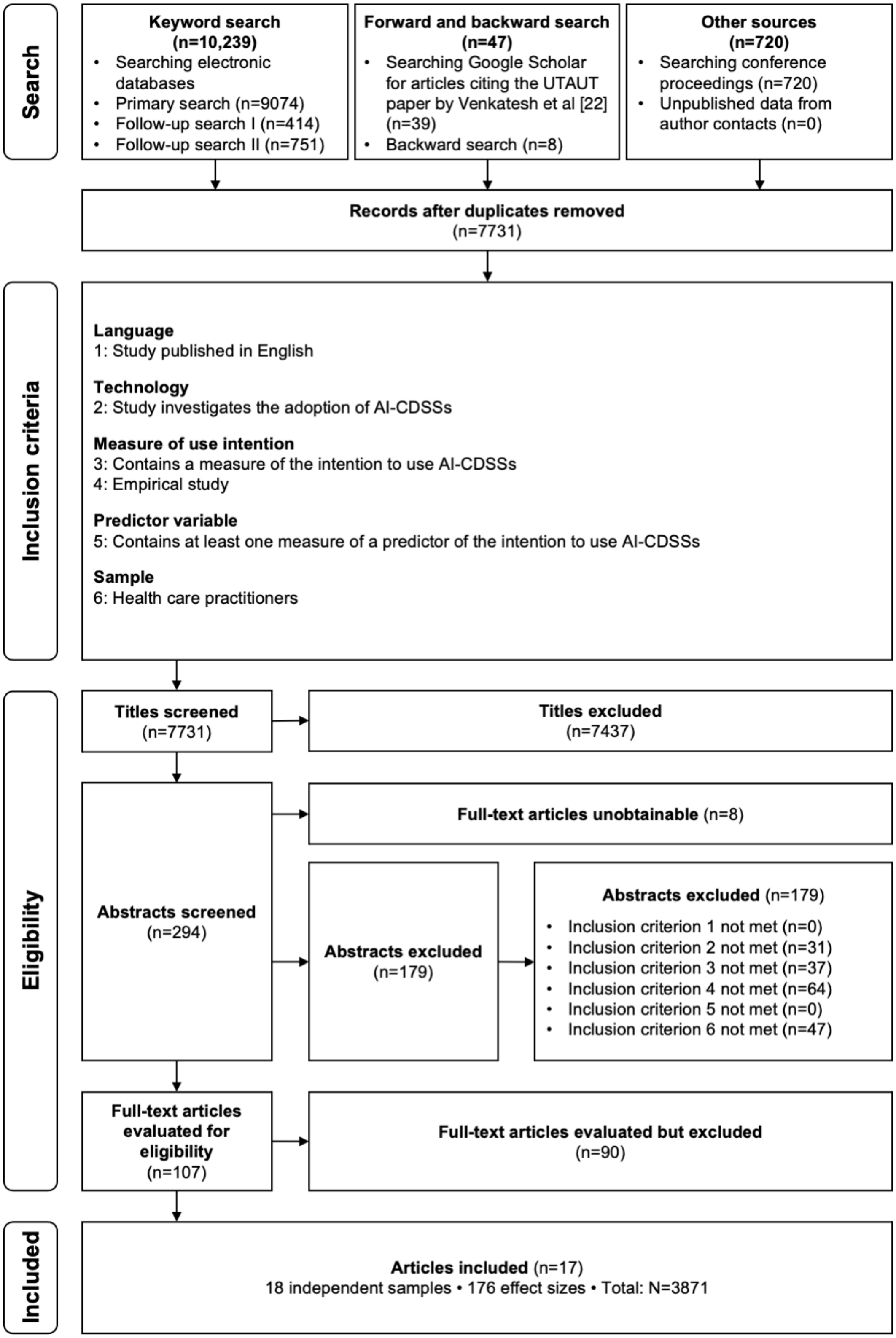

Figure S2. Age as a moderator of the social influence–use intention relationship

The solid line represents the estimate of the linear relationship (B) between sample mean age and the correlation between social influence and use intention. Individual points represent the observed correlation in each study, scaled in terms of their sample size (n).

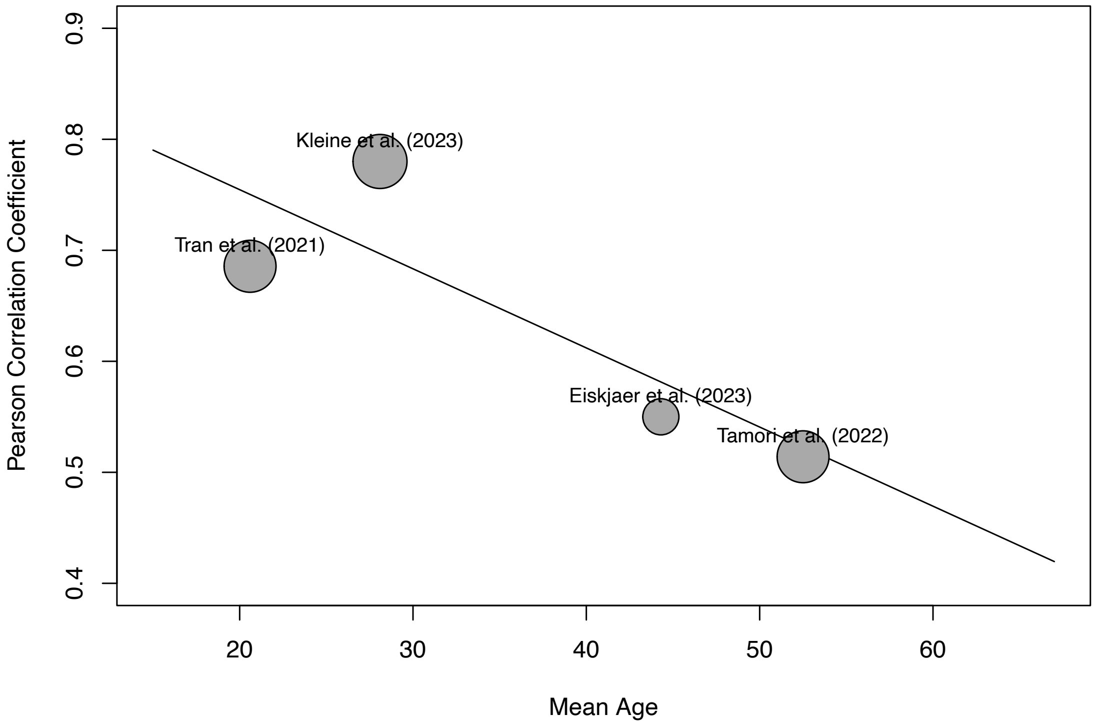

Figure S3. Results of cumulative meta-analyses

The dots indicate sample size-weighted and reliability-corrected correlations (r_c), intervals marked by vertical lines indicating 95% confidence intervals of r_c, and intervals marked by horizontal lines indicating 80% credibility intervals of r_c for each cumulative meta-analysis.

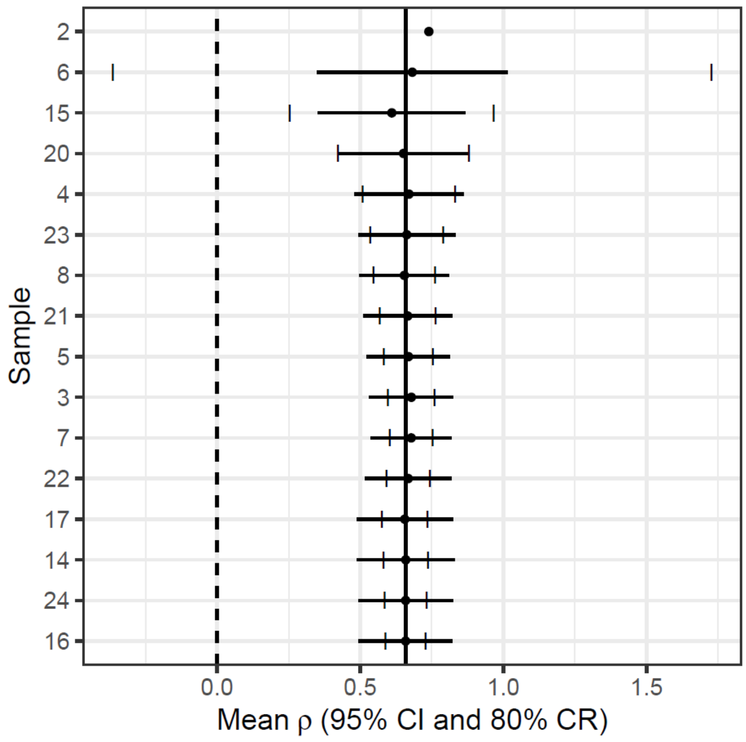

(a) *Performance expectancy*

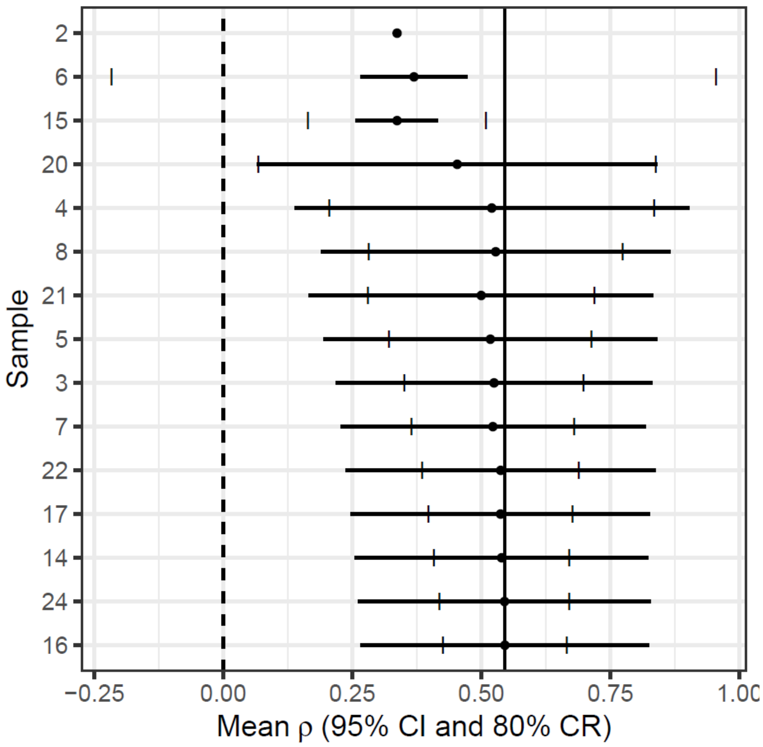

(b) *Effort expectancy*

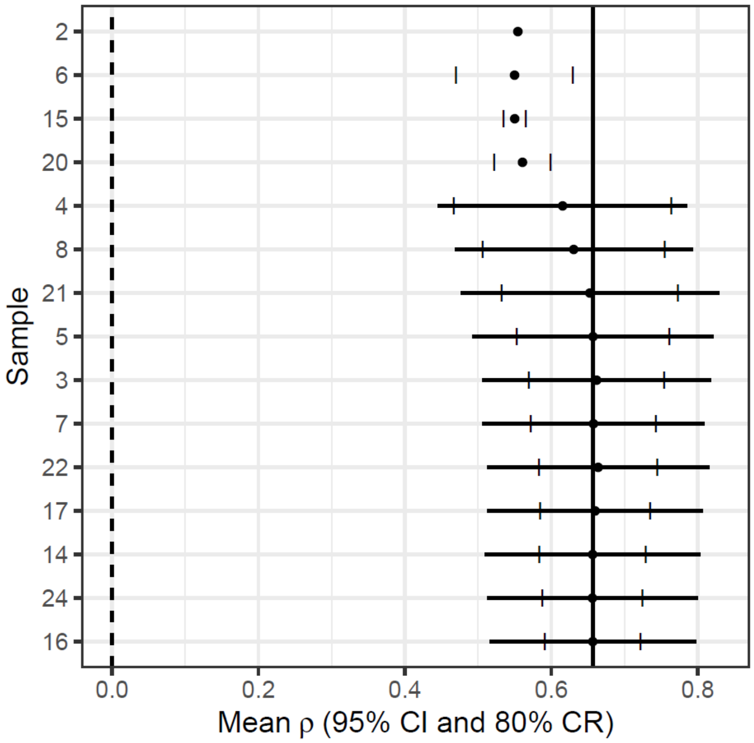

(c) *Social influence*

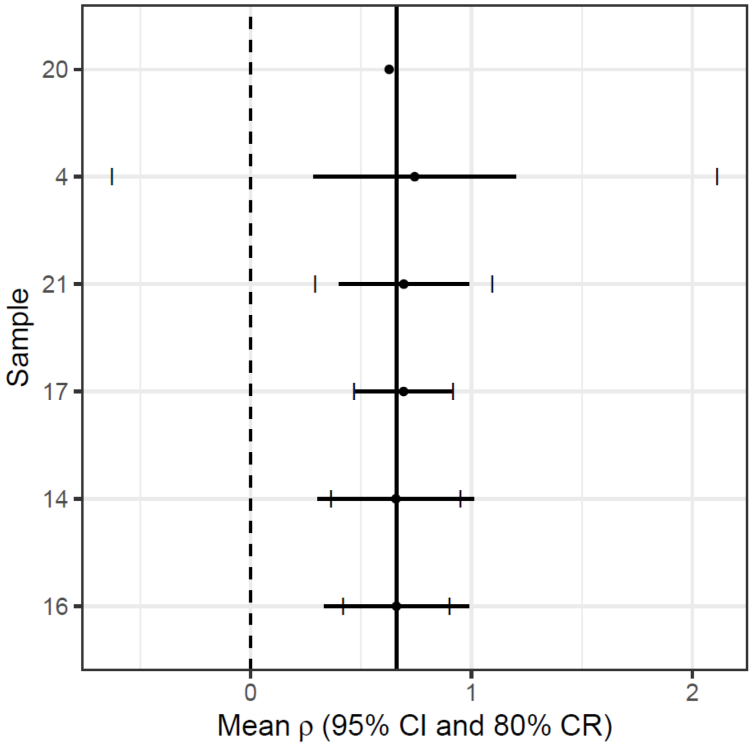

(d) *Facilitating conditions*

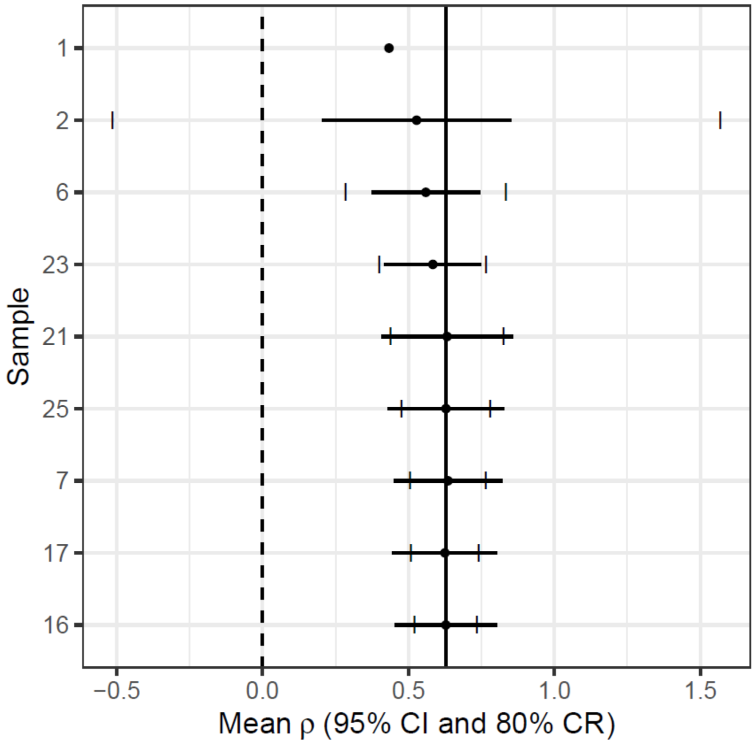

(e) *Attitude*

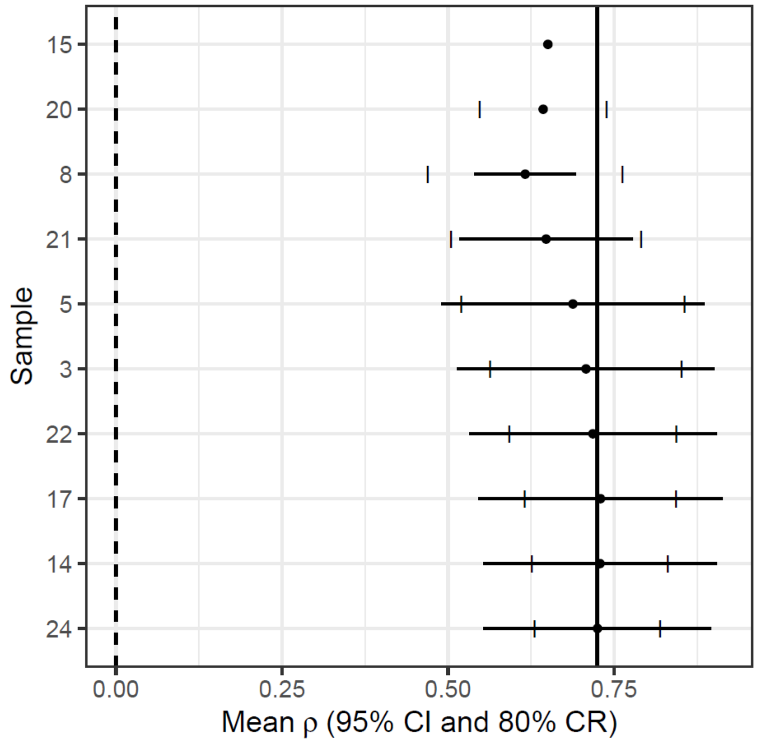

(f) *Trust*

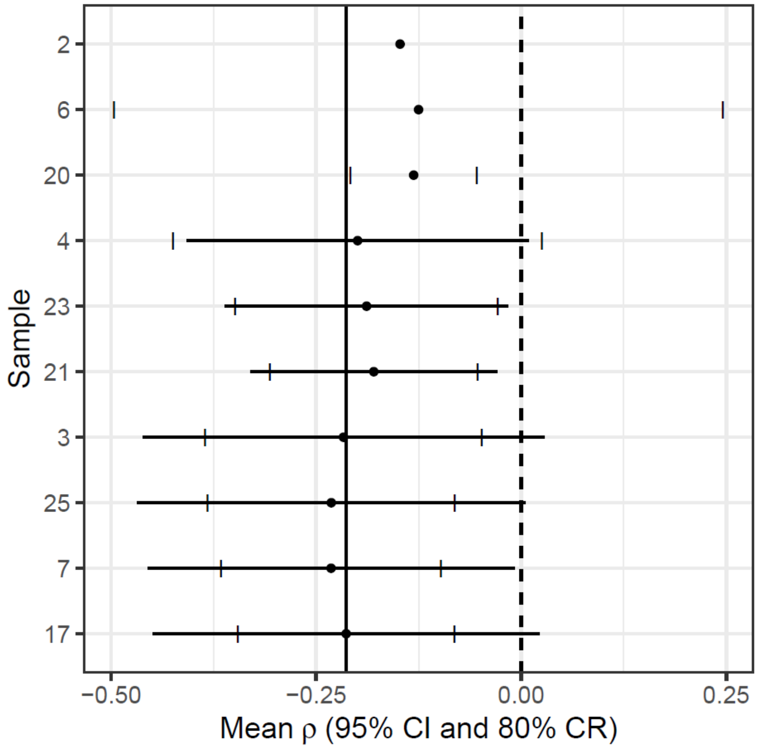

(g) *Perceived risk*

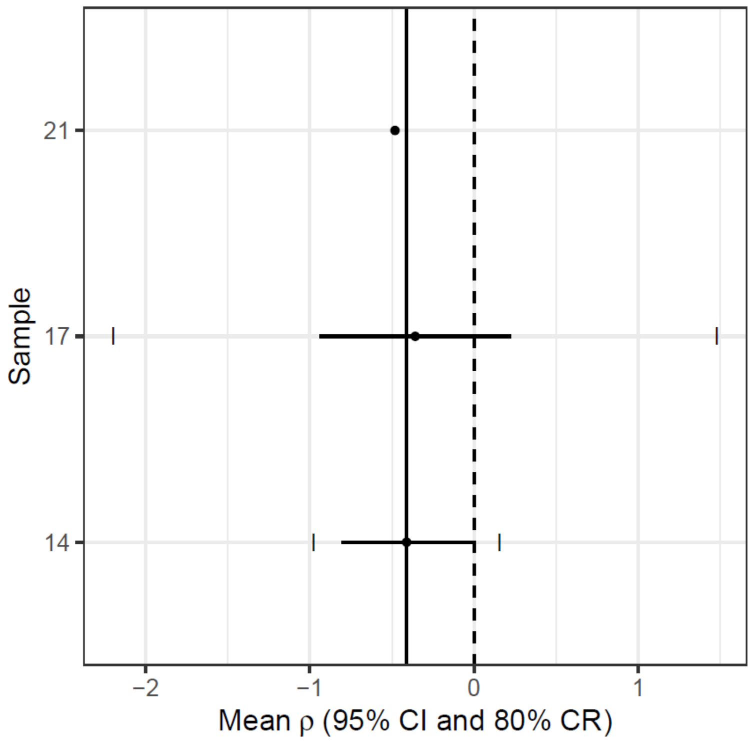

(h) *AI Anxiety*

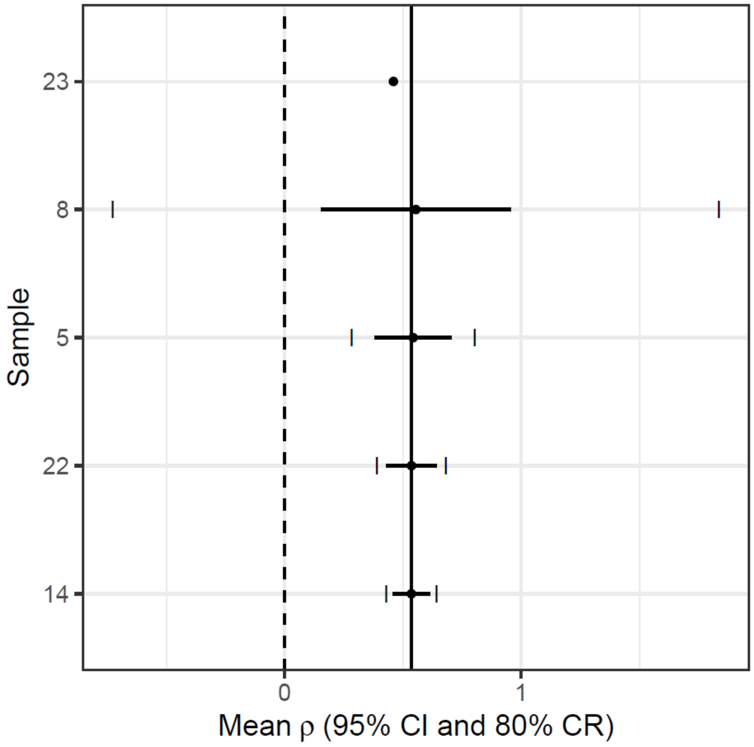

(i) *Innovativeness*

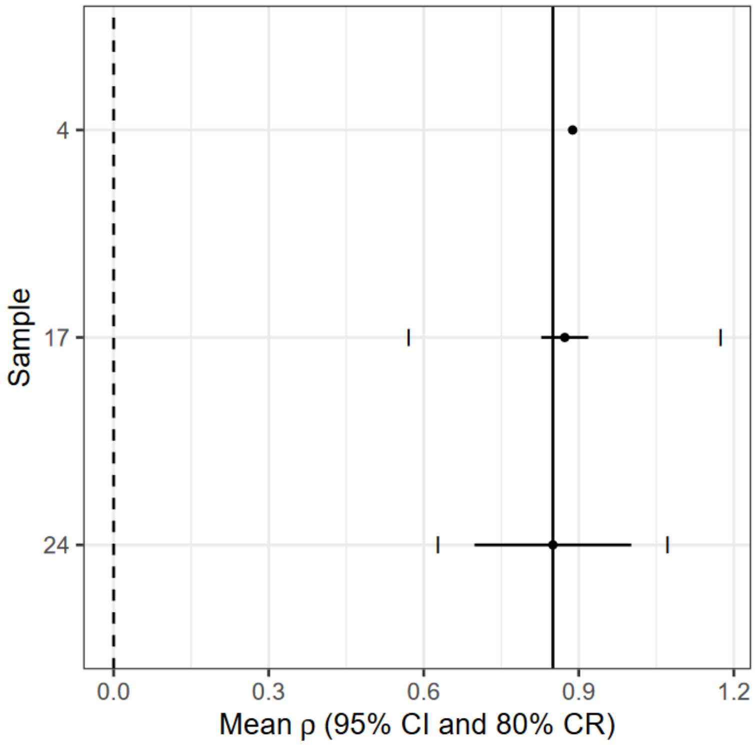

(j) *Actual use*
